# The Role of DNA Methylation Reprogramming During Sex Determination and Transition in Zebrafish

**DOI:** 10.1016/j.gpb.2020.10.004

**Published:** 2021-02-19

**Authors:** Xinxin Wang, Xin Ma, Gaobo Wei, Weirui Ma, Zhen Zhang, Xuepeng Chen, Lei Gao, Zhenbo Liu, Yue Yuan, Lizhi Yi, Jun Wang, Toshinobu Tokumoto, Junjiu Huang, Dahua Chen, Jian Zhang, Jiang Liu

**Affiliations:** 1 CAS Key Laboratory of Genome Sciences and Information, Beijing Institute of Genomics, Chinese Academy of Sciences / China National Center for Bioinformation, Beijing 100101, China; 2 State Key Laboratory of Membrane Biology, Institute of Zoology, Chinese Academy of Sciences, Beijing 100101, China; 3 University of Chinese Academy of Sciences, Beijing 100049, China; 4 State Key Laboratory of Molecular Developmental Biology, Institute of Genetics and Developmental Biology, Chinese Academy of Sciences, Beijing 100101, China; 5 Key Laboratory of Reproductive Medicine of Guangdong Province, the First Affiliated Hospital and School of Life Sciences, Sun Yat-sen University, Guangzhou 510275, China; 6 Integrated Bioscience Section, Graduate School of Science and Technology, National University Corporation Shizuoka University, Shizuoka 422-8529, Japan; 7 State Key Laboratory for Conservation and Utilization of Bio-Resources in Yunnan, Center for Life Sciences, School of Life Sciences, Yunnan University, Kunming 650091, China; 8 CAS Center for Excellence in Animal Evolution and Genetics, Chinese Academy of Sciences, Kunming 650223, China

**Keywords:** Germ cell, DNA methylation, Sex determination, Sex transition, Sexual plasticity

## Abstract

**DNA methylation** is a prevalent epigenetic modification in vertebrates, and it has been shown to be involved the regulation of gene expression and embryo development. However, it remains unclear how DNA methylation regulates sexual development, especially in species without sex chromosomes. To determine this, we utilized zebrafish to investigate DNA methylation reprogramming during juvenile **germ cell** development and adult female-to-male **sex transition**. We reveal that primordial germ cells (PGCs) undergo significant DNA methylation reprogramming during germ cell development, and the methylome of PGCs is reset to an oocyte/ovary-like pattern at 9 days post fertilization (9 dpf). When DNA methyltransferase (DNMT) activity in juveniles was blocked after 9 dpf, the zebrafish developed into females. We also show that Tet3 is involved in PGC development. Notably, we find that DNA methylome reprogramming during adult zebrafish sex transition is similar to the reprogramming during the sex differentiation from 9 dpf PGCs to sperm. Furthermore, inhibiting DNMT activity can prevent the female-to-male sex transition, suggesting that methylation reprogramming is required for zebrafish sex transition. In summary, DNA methylation plays important roles in zebrafish germ cell development and **sexual plasticity**.

## Introduction

Sex determination in animals has been one of the most fantastic mysteries in biology. In some species, sex is determined by sex chromosomes, while in other species no sex chromosomes are found in their genomes [1]. The mechanism of sex determination in these species is poorly understood. Even more interestingly, some adult animals can change their sex depending on their environmental conditions. For example, sexual fate in sea goldie can be regulated by the presence or absence of male fish within the population. When the dominant male dies in the group, one of the females will transition into a functional male [2], [3].

Many fishes, such as domesticated laboratory zebrafish strains, do not have sex chromosomes [4]. Prior to the sex determination stage during zebrafish germ cell development, juvenile zebrafish has an ovary-like gonad which contains early stage oocytes [5]. If these early stage oocytes continue to mature, zebrafish will develop into females. On the contrary, if these oocytes start to degenerate, then “juvenile ovary to testis” transformation can be observed, and zebrafish will develop into males [6], [7], [8]. It has been found that zebrafish sexual fate can be affected by environmental factors, such as endocrine hormones and temperature [9], [10], [11]. Additionally, a previous study has also reported that the germ cell number can influence sex determination in zebrafish [12]. In their natural environment, zebrafish sex transition can be observed. In the laboratory, aromatase inhibitor can induce female zebrafish to transform into males, even after they have reached sexual maturity [13]. However, the molecular mechanism for this sexual plasticity in adult zebrafish remains largely elusive.

DNA methylation plays important roles in gene expression and development [14], [15], [16], [17], [18], [19]. Two waves of DNA methylation reprogramming occur during mammalian early embryogenesis and primordial germ cell (PGC) development [20], [21], [22], [23], [24], [25]. In mice, abrogating DNA demethylation in PGCs can lead to infertility [26], indicating the importance of DNA methylation reprogramming during germ cell development. In zebrafish, the DNA methylation reprogramming during early embryogenesis has been investigated, showing that sperm but not oocyte DNA methylome is inherited by the offspring [27], [28]. Nevertheless, the role of DNA methylation during zebrafish sex differentiation and transition remains largely unknown.

To address these issues, we systematically analyzed the DNA methylome and transcriptome during zebrafish germ cell development and female-to-male transition. Our study provides mechanistic insights into zebrafish sexual development.

## Results

### A unique DNA methylome reprogramming during zebrafish germ cell development

**Figure 1:**
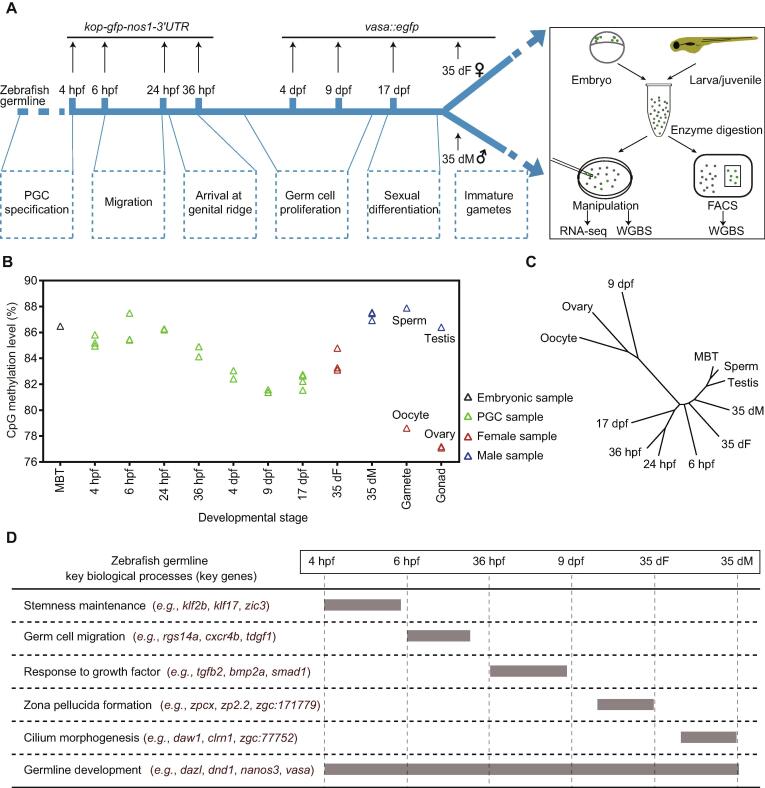
**Analysis of the DNA methylation dynamics during zebrafish germ cell development reveals a unique reprogramming pattern** **A.** The schematic diagram for zebrafish germ cell developmental stages in this study. Two transgenic strains were used to track the development of germ cells in zebrafish. Dotted boxes indicate the major developmental processes of germ cells at different stages. **B.** Global DNA methylation levels during zebrafish germ cell development. The methylation level was calculated as the average across all covered CpGs in the genome. DNA methylation data of zebrafish sperm, oocyte, and MBT stages were obtained from GEO: GSE44075. **C.** A dendrogram showing the clustering of DNA methylomes during zebrafish germ cell development. The R package ‘ape’ was used to perform hierarchical clustering analysis with ‘euclidean’ distance and the ‘complete’ clustering method (500 bp bin for each unit). **D.** A brief summary of expression patterns of key genes and key biological processes during zebrafish germ cell development. PGC, primordial germ cell; hpf, hours post fertilization; dpf, days post fertilization; dF, days post fertilization female; dM, days post fertilization male; FACS, fluorescence-activated cell sorting; WGBS, whole genome bisulfite sequencing; MBT, midblastula transition.

To investigate the dynamics of the DNA methylome during zebrafish germ cell development, we collected the samples of 9 representative stages during PGC development and germ cell differentiation. We first generated a *kop-gfp-nos1-3’UTR* transgenic strain to collect early PGCs [29], including 4 hours post fertilization (4 hpf) PGCs at specification stage, 6 hpf PGCs at migration stage, and 24/36 hpf PGCs arriving at genital ridge (Figure 1A). *kop*-GFP expression cannot be detected in later germ cell development stages, but *vasa* can mark later germ cells [30], [31], [32]. Thus, we generated a *vasa::egfp* transgenic strain to trace later germ cells. We collected PGCs at 4 days post fertilization (dpf), 9 dpf, and 17 dpf from juvenile gonads, germ cells (stage-1B oocytes [33] with 30–50 μm diameter) from 35 dpf female zebrafish (denoted as 35 dF germ cells), and germ cells (GFP-positive cells with less than 10 μm diameter) from 35 dpf male zebrafish (denoted as 35 dM germ cells) (Figure 1A, Figure S1A). Zebrafish sex differentiation begins at 17 dpf [34]. In this study, germ cells from 4 hpf to 17 dpf stages are uniformly described as “PGCs”. Micromanipulation and fluorescence-activated cell sorting (FACS) methods were employed to isolate the germ cells (Figure S1B; see details in Materials and methods). Highly purified germ cells were used to prepare DNA methylation libraries by a low-input whole genome bisulfite sequencing (WGBS) method [35] with at least two biological replicates (Table S1). CpGs covered by at least 3 reads were used for subsequent analysis. Our data show that CpG density is anti-correlated with DNA methylation level across all stages (Figure S2A). Most of the genomic elements are hypermethylated, except for 5’UTRs and promoter CpG islands (CGIs) (Figure S2B). In contrast, half of long non-coding RNAs (lncRNAs) [36] show intermediate or low methylation levels, which differ from tRNA and rRNA methylation levels (Figure S2C).

Next, we examined the average methylation levels (aMLs) of PGCs at all examined stages. The global methylation levels do not substantially change for the early PGCs from 4 hpf to 24 hpf, which are similar to that of midblastula transition (MBT) embryos (Figure 1B). This observation is similar to the results from a previous study [37]. Intriguingly, our data further show that after PGCs arrive at the genital ridge, the aML of PGCs at 36 hpf starts to decrease and reaches the lowest point at 9 dpf (aML = 81%) which is comparable to that of oocytes (aML = 79%) and ovaries (aML = 77%). The hierarchical clustering analysis of DNA methylomes also shows that 9 dpf PGCs, oocytes, and ovaries cluster together, separated from the other stages (Figure 1C). This is different from a previous study reporting that PGCs which migrate into gonads maintain a stable methylome pattern [38]. Furthermore, the aML of 35 dM germ cells increases dramatically to a level similar to that of sperm. On the contrary, the aML of 35 dF germ cells is lower than that of 35 dM germ cells (Figure 1B). In summary, a unique reprogramming of DNA methylome occurs during zebrafish germ cell development, which is distinct from that in mammals [39].

### Transcriptome analysis reflects the cellular processes of zebrafish germ cell development

Besides DNA methylome, we also performed RNA-seq in zebrafish germ cells (Table S2). First, we identified the stage-specific expressed genes during germ cell development (Figure S3A; Table S3). Gene ontology (GO) enrichment analysis shows that 6 hpf expressed genes are enriched for somitogenesis and germ cell migration (Figure S3A), such as *rgs14a* (Figure 1D, Figure S3B). This result is consistent with findings showing that germ cells at 6 hpf undergo the process of migration. Our data also show that pluripotency genes such as *zic3* and *klf17* are especially expressed at the stages of 4 hpf and 6 hpf (Figure 1D, Figure S3B), suggesting that PGCs at 4 hpf and 6 hpf show more stemness. A significant number of genes are expressed in both 35 dF and 35 dM germ cells, but many genes are only highly expressed in 35 dF germ cells or 35 dM germ cells. Further GO analyses show that 35 dF germ cell-specific expressed genes are enriched in the processes of egg coat formation and binding of sperm to zona pellucida (Figure S3A), such as *zp2.2* (Figure 1D, Figure S3B), reflecting the character of oocytes. Notably, *zar1l*, *pou5f3*, and *ca15b* are only highly expressed in 35 dF germ cells but not in 35 dM germ cells. These genes may function specifically in female germ cells (Figure S3B). The 35 dM germ cell-specific expressed genes are enriched in cilium assembly (*e.g.*, *daw1*) (Figure 1D; Figure S3A and B), which aligns with the knowledge that the flagellum of sperm is a modified cilium. In addition, germ cell genes, such as *piwil* and *tdrd* family genes, show limited expression in early PGCs but highly express at the later stages (Figure S3B). However, genes like *vasa* and *dnd1*, remain highly expressed throughout the whole development period examined (Figure 1D, Figure S3B). Taken together, gene expression can reflect the general cellular processes of zebrafish germ cell development (Figure 1D).

### DNA methylation reprogramming plays roles in zebrafish germ cell development

**Figure 2:**
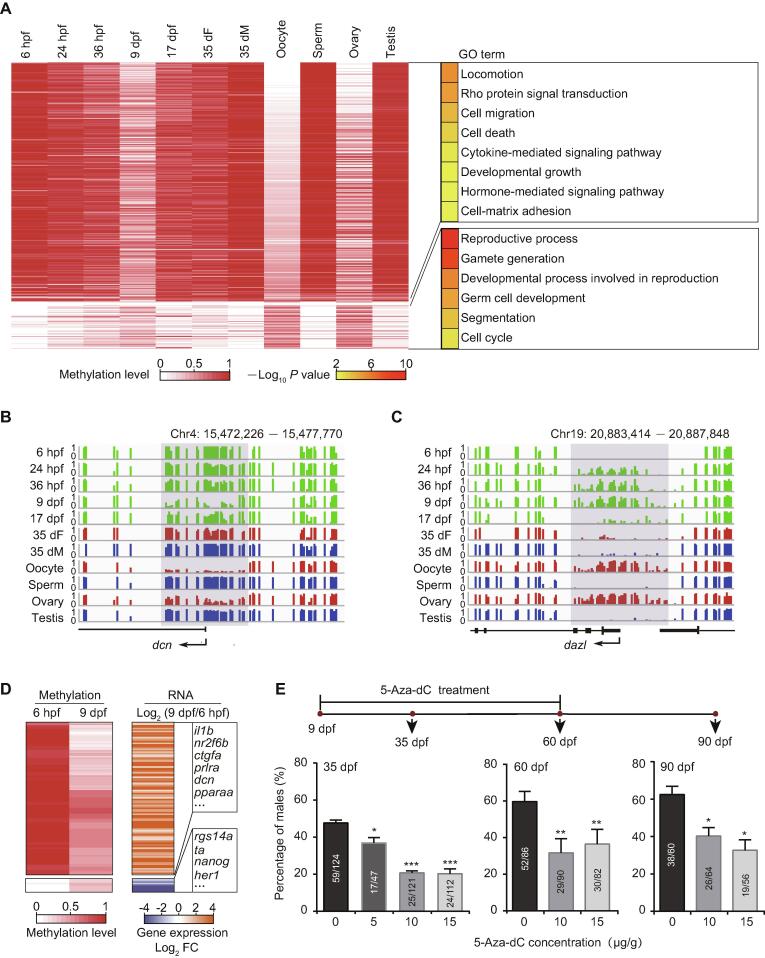
**The DNA methylation reprogramming is associated with zebrafish sexual development** **A.** Heatmap of DNA methylation levels for DMPs. These DMPs are classified into groups by PAM method. Corresponding GO terms are shown in the right panel and the color represents the enrichment statistical significance. Promoters are defined as 300 bp upstream and downstream of TSSs for each gene (*n* = 385). **B.** Snapshot for DNA methylation at 9 dpf-specific hypomethylated promoter of the gene *dcn.* Y axis represents DNA methylation level from 0 to 1. Each vertical line represents one CpG site. Dynamic regions around the promoter are highlighted in gray. **C.** Snapshot for DNA methylation at 9 dpf-specific hypermethylated promoter of the gene *dazl*. **D.** Heatmap of DNA methylation level (left) and gene expression FC (right) for genes with DMPs at 6 hpf and 9 dpf. Only genes with |log_2_ FC| > 1 are included. **E.** Sex ratio within zebrafish populations at 35 dpf, 60 dpf, and 90 dpf after treatment with 5-Aza-dC at different concentrations. The data are represented by mean ± SEM. Statistical significance was calculated by one-way ANOVA. *, *P* < 0.05; **, *P* < 0.01; ***. *P* < 0.001. DMP, differentially methylated promoter; PAM, Partitioning Around Medoid; TSS, transcriptional start site; GO, Gene ontology; FC, fold change; 5-Aza-dC, 5-Aza-2’-deoxycytidine.

Promoters are vital regulatory elements for gene transcription [40]. DNA methylation in promoters can regulate gene expression [41]. Therefore, we focused on the DNA methylation dynamics at promoter regions. We performed clustering analysis based on differentially methylated promoters (DMPs) (Figure 2A). Our data show that the stage-specific hypomethylated promoters at the 9 dpf stage compared to the other developing stages are also hypomethylated in oocytes/ovaries, and the stage-specific hypermethylated promoters at the 9 dpf stage are hypermethylated in oocytes/ovaries as well. These results indicate that the DNA methylation pattern of 9 dpf PGCs at promoters is similar to that of the oocytes/ovaries.

Further, GO enrichment analysis shows that genes with hypomethylated promoters in oocytes and 9 dpf PGCs are enriched in developmental growth, hormone-mediated signaling pathway, and cell migration (Figure 2A; Table S4), such as *myoc* (Figure S4A) and *dcn* (Figure 2B; regulating cell adhesion and migration by binding to extracellular matrix molecules [42]). The result is consistent with the observation that the PGCs migrate and then start to proliferate in gonads between 6 hpf and 9 dpf. Our data show that stage-specific hypermethylated promoters in 9 dpf PGCs are enriched in the reproductive process and germ cell development (Figure 2A; Table S4). These promoters become hypomethylated states at 17 dpf, 35 dF, and 35 dM. The representative examples of germ cell development related genes, including *dazl*, *tdrd1*, and *ta,* are shown in Figure 2C, Figure S4B, and Figure S4C, respectively. We speculate that genes with hypermethylated promoters at 9 dpf could help PGCs avoid the precocious differentiation.

To examine the potential regulation of promoter methylation on gene expression during zebrafish PGC development, we also analyzed the relationship of differential gene expression and promoter methylation between 6 hpf and 9 dpf PGCs. Our results illustrate that gene expression usually increases when the promoter methylation level decreases, including genes involved in cell migration, developmental growth, and hormone-mediated signaling pathway (*e.g.*, *prlra*) (Figure 2D). By contrast, gene expression usually decreases when the promoter methylation level increases, including pluripotency genes (*e.g.*, *nanog*) (Figure 2D). These results suggest that the dynamics of DNA methylation from 6 hpf to 9 dpf can inhibit the pluripotency capability of PGCs and facilitate PGC migration and proliferation.

At 35 dpf, the zebrafish sex has been determined. Gonads of female zebrafish at 35 dpf contain immature oocytes (Figure S1A) and show expression of oocyte-related genes (Figure S3B). However, there are a large amount of DMPs between 35 dF germ cells and oocytes, indicating that DNA methylome of 35 dF germ cells is different from that of oocytes (Figure 2A). In contrast, the methylome pattern of 35 dM germ cells is quite similar to that of sperm (Figure 2A). These results suggest that the DNA methylome of sperm is nearly established as early as 35 dpf, whereas dramatic DNA methylation reprogramming is necessary for oocyte maturation after 35 dpf.

To further elucidate the role of DNA methylation in sex determination, we used 5-Aza-2’-deoxycytidine [5-Aza-dC; an inhibitor of DNA methyltransferases (DNMTs)] to block DNA methylation during zebrafish germ cell development. We utilized three doses of 5-Aza-dC (5 μg/g, 10 μg/g, and 15 μg/g) to treat the *vasa::egfp* transgenic strain from 9 dpf to 60 dpf, and raised the treated juvenile fish until 90 dpf. Then we checked the male sex ratio in each group (see details in Materials and methods). The male sex ratio in the treated group is lower than that in the control group (0 μg/g) from 35 dpf to 90 dpf (Figure 2E; Table S5), indicating that fish treated with DNA methylation inhibitor prefer to develop into females, which is consistent with a recent study [43]. These results suggest that DNA methylation plays a role during sex determination.

### Interfering *tet3* gene expression leads to the reduction of PGC numbers

**Figure 3:**
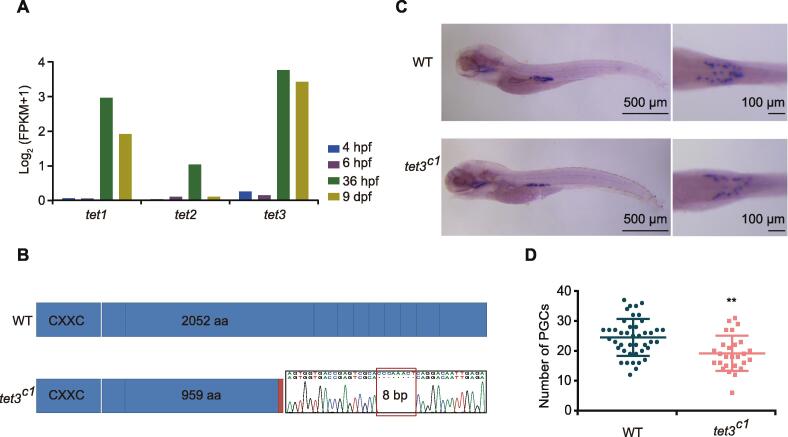
**The *tet3* mutation can cause the reduction of PGC numbers in zebrafish juveniles** **A.** Histogram of the gene expression levels of *tet1/tet2/tet3* in PGCs. Gene expression level was averaged from two biological replicates. **B.** Schematic diagram showing CRISPR/Cas9 editing in zebrafish *tet3* gene, with an 8 bp deletion in exon 3. **C.** Whole mount *in situ* hybridization showing expression of *vasa* mRNA in WT and *tet3^c1^* mutant fish at the 5 dpf stage. **D.** Number of PGCs at 5 dpf counted under stereoscopic microscope (WT, *n* = 43; *tet3^c1^*, *n* = 27). The data are represented by mean ± SD. *P* values are calculated by *t*-test. **, *P* < 0.01. WT, wild-type.

Our data show that there are more than 10,000 demethylated differentially methylated regions (DMRs) between the 36 hpf and 9 dpf stages (Figure S5A). Tet proteins were proposed to be involved in the DNA demethylation in mammalian embryos [44] and zebrafish phylotypic-stage embryos [45]. Our data show that *tet1* and *tet3* but not *tet2* are highly expressed in PGCs at the 36 hpf and 9 dpf stages (Figure 3A). To better define the role of Tet during germ cell development, we used the morpholino antisense oligonucleotide (MO) knockdown approach to target *tet1* and *tet3* in *vasa::egfp* transgenic embryos and validated the results by Western blot (Figure S5B). The majority of embryos injected with double MOs (*tet1* MO + *tet3* MO) show developmental deformity and lethality (Figure S5C). The *tet1* MO embryos do not show abnormal phenotypes. By contrast, *tet3* MO individuals show much weaker fluorescence signals in gonads at 5 dpf and 9 dpf compared to the wild type (Figure S5D). Previous studies have reported that the germ cell number can affect sex determination in zebrafish [12]. Our results show that the number of PGCs in *tet3* MO juveniles is significantly reduced (Figure S5E).

Next, we introduced mutations in the zebrafish *tet3* gene by using the CRISPR/Cas9 method. The *tet3^c1^* allele is a mutation allele with an 8 bp deletion in exon 3. This deletion leads to a premature stop codon in exon 3 and the loss of catalytic domain in the C-terminal region (Figure 3B). Then, we counted the number of PGCs in *tet3^c1^* mutants at 5 dpf using stereoscopic microscopy by whole mount *in situ* hybridization with *vasa* probes (Figure 3C and D). We observed that *tet3^c1^* fish contain less *vasa*-positive cells compared to the wild type, indicating that interrupting the *tet3* gene can reduce PGC numbers in zebrafish. It is suggested that Tet3 is involved in the regulation of PGC development.

### Induction of female**-to-**male sex transition in zebrafish by treatment with aromatase inhibitor

**Figure 4:**
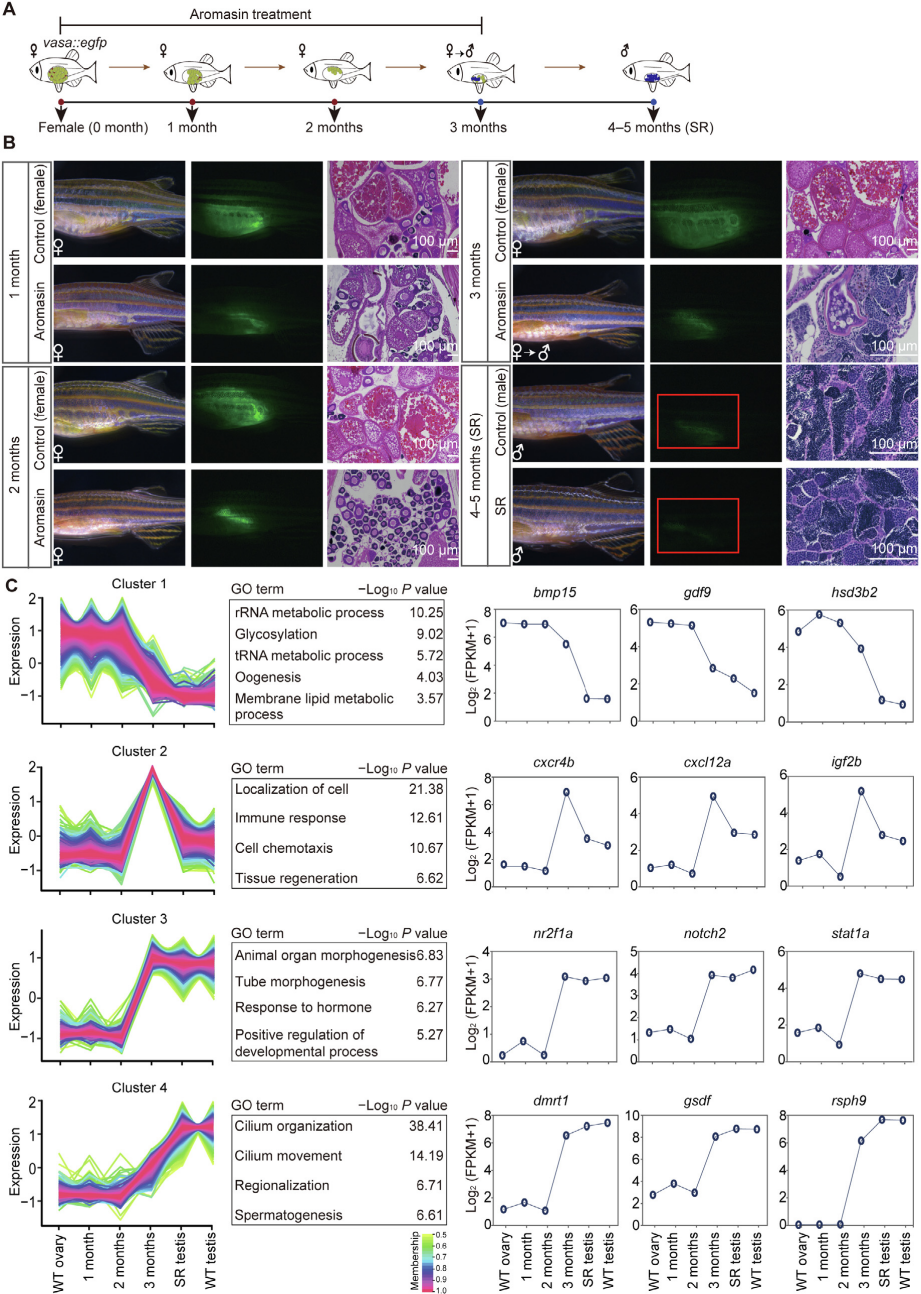
**Aromasin treatment can induce female**-**to**-**male sex transitionin adult zebrafish** **A.** The schematic diagram for zebrafish SR samples in this study. *vasa::egfp* transgenic zebrafish was used to induce sex transition by aromasin treatment. The samples include WT ovary, gonads from zebrafish treated with aromasin for 1 month, 2 months, or 3 months, SR testis, and WT testis. **B.** Observation of gonad morphological change in control female and aromasin-treated fish. Left, gonads under bright filed; middle, GFP fluorescence; right, H&E staining. Red boxes indicate the regions of weak fluorescence of testis. The scale bar in H&E staining indicates 100 µm. **C.** Time-course clustering analysis of gene expression patterns throughout zebrafish SR process. For the four distinct expression clusters, the left plots of each cluster show the gene expression pattern, the middle tables show the GO enrichment results, and the right plots show dynamics of representative gene expression levels. SR, sex-reversed; H&E, Hematoxylin and Eosin.

Inhibiting aromatase activity by aromatase inhibitor can revert adult zebrafish from female to male [13]. This female-to-male sex transition process provides us a chance to explore the underlying molecular mechanisms for adult sexual plasticity in this vertebrate. We used the aromatase inhibitor aromasin (exemestane) to induce female-to-male sex transition in *vasa::egfp* transgenic zebrafish (Figure 4A and B; see details in Materials and methods). Our results show that the EGFP fluorescence intensity within adult gonads is gradually decreased after aromasin treatment, suggesting that the female germ cells start degenerating (Figure 4B). Histological staining also shows that the number of female germ cells is gradually reduced (Figure 4B). After aromasin treatment for 3 months, the gonads exhibit an intermediate transition state containing both degenerated oocytes and emerged male germ cells. We then stopped the aromasin treatment and found that the treated female fish would develop a testis-like gonad in the next 1 or 2 months (Figure 4B). We further mated the sex-reversed fish with wild-type females. Our result shows that the sperm from sex-reversed fish is able to successfully fertilize and produce normal embryos (Figure S6A).

We then performed RNA-seq to investigate the dynamics of gene expression during female-to-male sex transition (Table S2). Using the time-course gene expression clustering analysis (see details in Materials and methods), we identified four dynamic gene clusters (Figure 4C; Table S6). Our data show that the expression levels of female sexual development genes (cluster 1, *e.g.*, *bmp15* and *hsd3b2*) are down-regulated during the sex transition process (Figure 4C). It is worth noting that the 3-month stage is the intermediate transition stage (Figure 4B). Genes in cluster 2 display uniquely high expression at this stage but low expression in the ovary and the sex-reversed/wild-type testis. These genes are enriched in the tissue regeneration process (*e.g.*, *igf2b*), immune response, and localization of cell (*e.g.*, *cxcr4b* and *cxcl12a*) (Figure 4C). We also found genes which become highly expressed at the intermediate transition stage and maintain high levels in the sex-reversed/wild-type testis (cluster 3). These genes are enriched in organ morphogenesis (*e.g.*, *notch2*) and hormone response (Figure 4C). Male sexual development genes (cluster 4, *e.g.*, *dmrt1* and *gsdf*) exhibit high expression at the intermediate transition stage, and their expression levels are further up-regulated in the sex-reversed/wild-type testis (Figure 4C). In summary, during female-to-male transition, genes related to female sexual development reduce expression first, genes related to tissue regeneration, cell migration, and organ morphogenesis start expression at the intermediate transition stage, and genes related to male and sperm generation increase expression in the end.

### Significant genome-wide DNA methylome changes during zebrafish female**-to-**male sex transition

**Figure 5:**
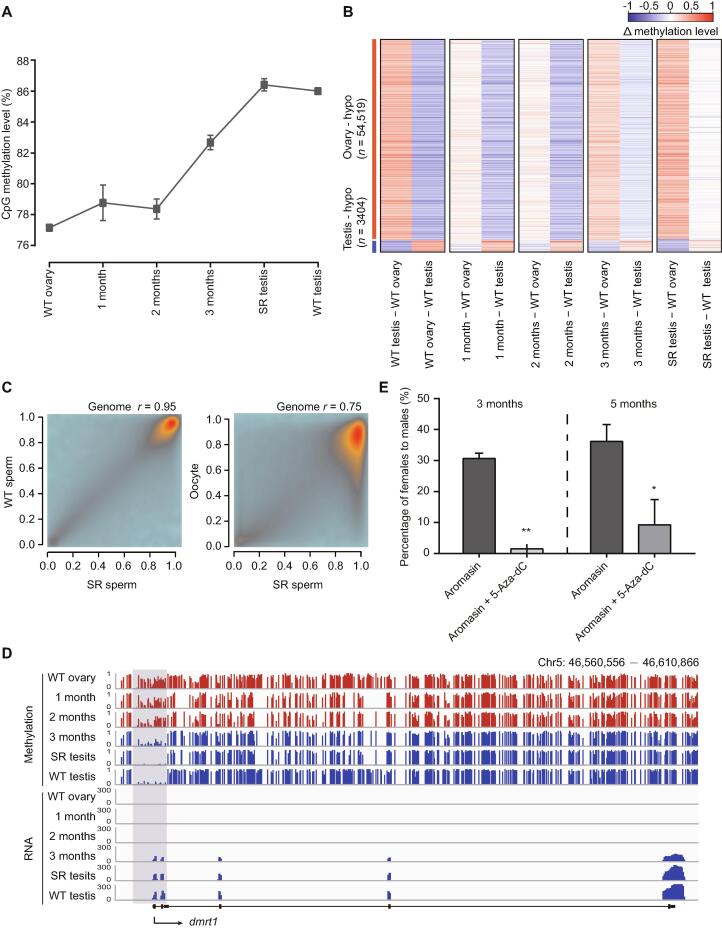
**DNA methylomeisresetfrom the female pattern to the male pattern during zebrafish sex transition** **A.** The global DNA methylation reprogramming during zebrafish sex transition. The methylation level was calculated as the average across all covered CpGs in the genome. The data are represented by mean ± SD. The samples include WT ovary, gonads from zebrafish treated with aromasin for 1 month, 2 months, or 3 months, SR testis, and WT testis. **B.** The dynamic changes for DMRs between zebrafish ovary and testis during zebrafish sex transition. The Δ methylation heatmap displays the difference in DNA methylation level between two consecutive stages during zebrafish sex transition. ‘*n*’ represents the number of ovary-specific hypo regions or testis-specific hypo regions. **C.** The correlation analysis of CpG methylation levels between SR sperm and WT sperm or oocyte. The Pearson correlation coefficients (*r*) is shown on the top. **D.** Snapshot of DNA methylation and gene expression tracks for the sex determination gene *dmrt1* in WT ovary, gonads from zebrafish treated with aromasin for 1 month, 2 months, or 3 months, SR testis, and WT testis. The promoter of *dmrt1* is defined as regions 1000 bp upstream and downstream of the TSS. Dynamic regions around the promoter are highlighted in gray. **E.** Sex ratio within populations at 3 months (aromasin only, *n* = 36; aromasin + 5-Aza-dC, *n* = 47) and 5 months (sex-reversed stage; aromasin only, *n* = 36; aromasin + 5-Aza-dC, *n* = 38) after drug treatment. Fish were treated with aromasin only / aromasin + 5-Aza-dC for 3 months, and then raised till 5 months. Three independent experiments were performed for each drug treatment group. The data are represented by mean ± SEM. Statistical significance was calculated by unpaired two-sided *t*-test. *, *P* < 0.05; **, *P* < 0.01. DMR, differentially methylated region; hypo, hypomethylated.

Our results have shown that DNA methylation is tightly associated with germ cell development in juvenile zebrafish (Figure 2). To explore the role of DNA methylation in adult zebrafish sex transition, we sequenced DNA methylomes of gonads during the sex transition process, including wild-type ovary/testis, gonads treated with aromasin for 1 month, 2 months, or 3 months, and fully sex-reversed testis with at least two independent biological replicates (Figure 4A, Figure S6B; Table S1). Our data show that the global DNA methylation level has a significant change during the female-to-male sex transition (Figure 5A), and the aML of gonads after 3-month aromasin treatment (aML = 83%) is between that of wild-type ovary (aML = 77%) and wild-type testis (aML = 86%) (Figure 5A and B, Figure S6C). When the treated females sexually revert to males, the aML of sex-reversed testis reaches a similar level to that of wild-type testis (Figure 5A and B, Figure S6C). The results above indicate that sperm from sex-reversed fish have the same functions as wild-type sperm (Figure S6A). We thus sequenced the DNA methylomes of sex-reversed sperm and wild-type sperm. Our data show that the DNA methylome of sperm from sex-reversed fish is similar to that of sperm from wild-type fish, but different from that of oocytes (Figure 5C). For example, *dnmt6* is low-methylated in oocyte, but it becomes high-methylated in sex-reversed sperm, which is similar to the state in wild-type sperm (Figure S6D); *sycp3l* is high-methylated in oocyte, but it is low-methylated in both sex-reversed and wild-type sperm (Figure S6E).

Our data illustrate that several well-known sex determination genes show different methylation patterns at their promoters between ovaries and testes (Figure S7A and B; Table S7). We investigated the relationship between transcription and methylation statuses for these known sex determination genes during sex transition. For example, a well-defined sex determination gene *dmrt1*, a key regulator of sex determination which is necessary for male sexual development in vertebrates [46], [47], [48], [49], has a male-specific hypomethylated promoter and starts to be highly expressed from 3 months onwards (Figure 5D). In addition, *cyp19a1a*, a critical gene in promoting ovary development in fish [50], shows a low methylation level at the promoter region in ovaries, but gradually reprograms to a high methylation level at the later stages (Figure S7C).

We next compared the DNA methylation dynamics between the adult sex transition process and juvenile germ cell development in zebrafish. The dynamics of methylation levels during female-to-male sex transition is very similar to that from 9 dpf PGCs to testis during normal development (Figure S7D and E). It suggests that the ability of female gonads to transform into male gonads may be due to similar methylome patterns between female gonads and 9 dpf PGCs with bipotential differentiation capacity.

Furthermore, we wanted to know the role of DNA methylome during the zebrafish sex transition process. We added 5-Aza-dC (35 μg/g) to examine its effect on aromasin-induced female-to-male sex transition (see details in Materials and methods). After drug treatment for 3 months, 31% of total females treated only with aromasin start transitioning into males, as these fish have both ovary and testis, while almost none of females treated with both 5-Aza-dC and aromasin undergo the transition. We then stoped the drug treatment and raised the treated fish in the recirculation system till 5 months. We found that 36% of total females transition into males in the aromasin-only treatment group, whereas merely 9% of females transition into males in the aromasin + 5-Aza-dC treatment group (Figure 5E; Table S5).

In summary, our results indicate that blocking DNA methylation can prevent sex transition in zebrafish, suggesting that DNA methylation dynamics is essential for sex transition in adult zebrafish.

## Discussion

Genome-wide erasure of DNA methylation during germ cell development has been documented in mammals [39], [22], [23], [24], [25], but it was limitedly reported in zebrafish germ cells. A recent study generated low-depth DNA methylome data of zebrafish PGCs after 24 hpf [38]. Low depth and coverage of the DNA methylome data hinder the understanding of germ cell development. They reported that zebrafish did not undergo significant reprogramming during PGC development. In contrast, we generated high-depth DNA methylome data for zebrafish, including early PGCs and later germ cells, using modified library generation methods at a single-base resolution [35]. Our results show that zebrafish PGCs undergo significant DNA methylation reprogramming during germ cell development. More importantly, the observation that the methylome of PGCs is reset to an oocyte/ovary-like pattern at 9 dpf suggests that zebrafish PGCs at 9 dpf have the bipotential to differentiate into male or female germ cells. It is interesting to note that the DNA methylation reprogramming pattern during normal sexual development from 9 dpf PGCs to male testis is similar to that during aromasin-induced female ovary-to-male testis transition. Thus, we infer that the 9 dpf-like bipotential DNA methylation pattern in female gonads is the reason why the female gonads are able to transform into the male gonads. The role of DNA methylation in sex transition has not been revealed previously. In this study, we demonstrate that DNA methylation reprogramming is required for the sex transition in zebrafish, by inhibiting the DNA methylation reprogramming with 5-Aza-dC treatment.

During artificially induced zebrafish female-to-male sex transition, our results indicate that there exists DNA methylation dynamics for vital sex determination genes, including *dmrt1* and *cyp19a1a*, which shows a similar trend in blue head wrasses and half-smooth tongue sole [51], [52]. These results suggest that DNA methylation may have a conserved regulatory role in fish sex determination.

A recent study has reported that H3K27me3 plays roles in turtle sex determination [53]. The interplay of DNA methylation and other epigenetic modifications for zebrafish sex determination is worthy of further study.

Taken together, our research provides insight into the molecular mechanism and understanding of the mystery of sex transition, and we reveal that the DNA methylome reprogramming plays a key role in zebrafish germ cell development and female-to-male sex transition.

## Materials and methods

### Experimental model and subject details

All animal maintenance and experimental procedures were carried out according to the guidelines of the Institutional Animal Care and Use Committee (IACUC) of the Beijing Institute of Genomics, Chinese Academy of Sciences (CAS) / China National Center for Bioinformation, Beijing, China. All zebrafish used in this study were maintained at 28 °C with a light/dark cycle of 14 h/10 h.

### Generation of the transgenic zebrafish lines

For the generation of the *kop-gfp-nos1-3’UTR* transgenic  zebrafish line (AB strain based), *kop-gfp-nos1-3’UTR* sequence was cloned into T2ASAd vector. The purified T2ASAd plasmid was injected into the cytoplasm of 1-cell stage zebrafish embryos with *Tol2* transposase mRNA. Female fish carrying the transgene were identified by screening of GFP expression in early PGCs.

For the generation of the *vasa::egfp* transgenic zebrafish line (AB strain based),  the *vasa::egfp* fragment was released from the *Xba*I and *Sfi*I restriction sites of pK4 vector [32], and then the purified fragment was injected into the cytoplasm of 1-cell stage zebrafish embryos. Injected embryos were screened for EGFP signals under a fluorescence microscope as founder candidates and cultured to sexual maturity. Female founder candidates were mated with wild-type males and screened for the production of eggs with EGFP signals.

### Sample collection and preparation of single cell suspension

For collection of 4 hpf and 6 hpf PGCs, *kop-gfp-nos1-3’UTR* transgenic zebrafish embryos were washed twice with ice-cold 1× DPBS (Catalog No. 14190144, Gibco, Waltham, MA), and chorions were carefully removed with sharp tweezers. 1 ml DPBS with 0.5% BSA was added to the collected embryos, and gentle pipetting was performed until all embryos were dissociated to single cells. All steps were operated on ice.

For collection of 24 hpf and 36 hpf PGCs, *kop-gfp-nos1-3’UTR* transgenic zebrafish embryos were placed on dish and depigmented by treating them with 1-phenyl 2-thiourea (PTU; catalog No. P7629, Sigma, St. Louis, MO). Chorions were removed with pronase for 10 min, and embryos were washed twice with ice-cold DPBS. Embryo yolks were removed with 1 ml ice-cold deyolking buffer (55 mM NaCl, 1.8 mM KCl, 1.25 mM NaHCO_3_). Then 0.25% trypsin (Catalog No. 25200056, Gibco) solution was added, and samples were incubated at 30 °C for 15 min followed by being washed with DPBS and centrifuged at 400 *g* for 3 min at 4 °C. Then isolated single cells were re-suspended in DPBS containing 5% EDTA.

For collection of 4 dpf and 9 dpf PGCs, *vasa::egfp* transgenic zebrafish juvenile heads and tails were removed, and body parts containing gonads were dissected into D-hanks (Catalog No. CC0023, Leagene, Beijing, China) solution, then transferred into 0.25% trypsin and incubated at 30 °C for 40 min. Gentle pipetting was performed for several times. The reaction was quenched with 10% fetal bovine serum (FBS), and individual cells were washed with DPBS and centrifuged at 400 *g* for 3 min at 4 °C. The cell pellet was re-suspended in DPBS with 0.5% BSA.

For collection of 17 dpf PGCs, 35 dF germ cells, and 35 dM germ cells, gonads from individual zebrafish were isolated and separated from surrounding tissues in D-hanks solution with the help of a dissecting fluorescence microscope. Immature females and males were determined via GFP intensity of *vasa::egfp* transgenic zebrafish under a fluorescence microscope. Gonads exhibiting high and low GFP fluorescence were classified as ovaries and testes, respectively [7]. The collected gonads were dissociated with 0.25% trypsin at 30 °C for 10 min. 10% FBS was added for termination of trypsin as described above. 35 dM germ cells (potential spermatocytes with less than 10 μm diameter) were centrifuged at 400 *g* for 3 min at 4 °C, while 35 dF germ cells (the stage-1B oocytes [33] with 30–50 μm diameter) were centrifuged at 100 *g* for 1 min at 4 °C to avoid cell membrane breakdown.

For each sample, GFP-positive cells were manually picked using micromanipulator system under the fluorescence microscope or were purified by FACS (BD FASAria II, BD Bio-sciences, Franklin Lakes, NJ). For FACS, the cell suspension was filtered with 30 μm Pre-Separation Filters (Catalog No. 130-041-407, Miltenyi Biotec, Köln, Germany) to remove cell aggregates before sorting. Cell purity was checked based on fluorescence under microscopy.

### Low-input bisulfate-seq library preparation for zebrafish germ cells

DNA methylation libraries of zebrafish germ cells were prepared with the low-input “One Tube” method as described previously [35]. 50–1000 cells were lysed in 20 μl lysis buffer (20 mM Tris, 2 mM EDTA, 20 mM KCl, 1 mg/ml protease K) for 1.5 h at 56 °C, followed by protease K inactivation for 30 min at 75 °C. 30 μl nuclease free water and 0.5% spike-in un-methylated lambda DNA were added into the lysate. DNA was sheared into 400 bp fragments by Covaris S220 (Woburn, MA). Fragmented DNA was then concentrated to 30 μl and subjected to end repair by incubating with 5 μl end-repair enzyme mixture [3.5 μl T4 DNA ligase buffer (Catalog No. M0202, NEB, Boston, MA), 0.35 μl 10 mM dNTP, 1.15 μl NEBNext End Repair Enzyme Mix (Catalog No. E6050, NEB)] for 30 min at 20 °C followed by heat-inactivation for 30 min at 75 °C. After end repair, 5 μl of dA-tailing mixture [0.5 μl T4 DNA ligase buffer, 1 μl Klenow Fragment  (3′→5′ exo-) (Catalog No. M0212, NEB), 0.5 μl 100 mM dATP, 3 μl nuclease free water] was added to the tube and incubated for 30 min at 37 °C followed by heat-inactivation for 30 min at 75 °C. Finally, 10 μl ligation mixture (1 μl T4 DNA ligase buffer, 0.5 μl 100 mM ATP, 1.5 μl 5 mM cytosine methylated Illumina adapter, 2 μl T4 DNA ligase, 5 μl nuclease free water) was added to the tube and incubated at 16 °C overnight. 100 ng carrier RNA was added into the tube. Bisulfite conversion reaction was performed with the EZ DNA Methylation-Gold Kit (Catalog No. D5006, Zymo Research, Irvine, CA) according to the manufacturer’s instructions. DNA was purified with column purification. The purified DNA was then amplified with 6 cycles of PCR by using KAPA HiFi HotStart Uracil + ReadyMix (Catalog No. kk2802, KAPA, Wilmington, MA). Amplified DNA was purified with 1 volume Ampure XP beads (Catalog No. A63881, Beckman Coulter, Brea, CA) to discard the short fragments and adapter-self ligations. 50 μl of XP beads was added to 50 μl of PCR product. Samples were mixed by pipetting and incubated at room temperature for 10 min, and keeping the beads on the magnet, samples were washed twice with 200 μl of 80% ethanol without mixing. Ethanol was then completely removed. Beads were left on the magnet for 5 min to allow the remaining ethanol to evaporate. DNA was eluted by adding 20 μl of ddH_2_O, mixing by pipetting, and incubating for 5 min. After separating on a magnet, the solution was transferred to a new tube. Then, another round of 6–8 cycles of PCR was performed to obtain sufficient molecules for sequencing. The number of PCR amplification cycles was determined according to the amount of 1 μl amplified DNA, which was evaluated using FlashGel System (Catalog No. 57063, Lonza, Switzerland). Lastly, 300–500 bp DNA fragments were selected using Ampure XP beads with 0.5× volume plus 0.65× volume, and then eluted in 15 μl ddH_2_O. DNA methylation libraries were sequenced on Hiseq2000 or Hiseq Xten platform (Illumina).

### Low-input RNA-seq library preparation for zebrafish germ cells

For low-input RNA-seq libraries, we picked up 20–50 GFP-positive germ cells at each stage by micromanipulator and transferred samples into 7 μl DPBS with a snap freeze in liquid nitrogen immediately. Reverse transcription using REPLI-g WTA Single Cell Kit (Catalog No. 150063, Qiagen, Hilden, Germany) was perfromed according to the kit’s standard protocol. The amplified cDNA was purified using 1 volume Ampure XP beads, and then fragmented to 200–400 bp by Covaris S220. NEBNext Ultra II DNA Library Prep Kit (Catalog No. E76455, NEB) was used for library construction according to manufacturer’s instruction. cDNA was end repaired and A-tailing by adding 7 μl NEBNext Ultra II End Prep Reaction Buffer and 3 μl NEBNext Ultra II End Prep Enzyme Mix. Samples were incubated in a thermal cycler at 20 °C for 30 min, 65 °C for 30 min, and finally cooled to 4 °C. Adaptor ligation was performed by adding 30 μl NEB Next Ultra II Ligation Master Mix, 1 μl NEBNext Ligation Enhancer, 0.5 μl 200 mM ATP, and 2.5 μl 25 mM cytosine methylated Illumina adapter. Sample were thoroughly mixed and incubated at 20 °C for 30 min. Adapter-ligated DNA was purified with 1 volume Ampure XP beads to discard the short fragments and adapter-self ligations, and then amplified with 6–7 PCR cycles. Sequencing was performed on Hiseq 2000 or Hiseq Xten platform (Illumina).

### Histology

For 35 dpf juveniles [females and males are approximately equal in length (around 1.7 ± 0.1 cm)] and sex-reversed adult fish, heads and tails were cut off. The middle body parts containing gonads were fixed in Bouin’s solution (Catalog No. HT10132, Sigma) overnight at 4 °C. After dehydration, samples were embedded in paraffin and sectioned at 10 μm thick. H&E staining (Catalog No. C0105S, Beyotime, Shanghai, China) was then performed on the sections.

### MO injection

To analyze the function of Tet, knockdown experiments were conducted by the injection of MOs, including *tet1* MO (5′-AGATGCACTGTCTGAAGGGCTAATA-3′) and *tet3* MO (5′-TGGGACCCAATTTCCATCTGGCCTT-3′), which were synthesized by Gene Tools (OR, USA). The standard control MO (5′-CCTCTTACCTCAGTTACAATTTATA-3′), also provided by Gene Tools, was used as the control.

Approximately 4 ng or 8 ng (double-MO injection, 4 ng each) MO solution was injected into the 1-cell stage *vasa::egfp* transgenic zebrafish embryos using a pressure microinjector. The phenotypes were observed under a stereoscope at different time points.

### Counting the number of PGCs in zebrafish germline

The number of PGCs was counted using the squash method at the 5th day and the 9th day as described previously [12]. Briefly, for 5 dpf larvae, we placed 5 μl water on the slide, put a larva in the water, and pressed a coverslip onto the sample. For 9 dpf zebrafish, larval heads and tails were removed, and then the body parts containing gonads were dissociated with 0.25% trypsin and incubated at 30 °C for 4 min. Next, we transferred each body part on the slide, and pressed a coverslip onto the sample. The number of PGCs was detected based on EGFP fluorescence under a fluorescence microscope.

### CRISPR/Cas9-mediated editing of ***tet3*** and genotyping

The *tet3* mutant zebrafish was generated by using CRISPR/Cas9 as described previously [54]. *tet3* gRNA (5′-ACCGAGTCGCACCCAAACTC-3′) and Cas9 mRNA were synthesized by *in vitro* transcription, and then injected into 1-cell stage embryos. For CRISPR/Cas9 efficiency identification and genotyping, the 24 hpf embryos and caudal fin tissues were dissociated with 50 mM NaOH at 95 °C for 10 min (vortexes twice) and then balanced with equal volume of 10 mM Tris-HCl (pH 8.0). The primers used for PCR were *tet3* cas9-S: 5′-AATAGCATGCCCAGGCTCAG-3′ and *tet3* cas9-AS: 5′-GACAGTACAGAGCTCCTCAGG-3′.

### Whole mount ***in situ*** hybridization

After the addition of PTU, the 5 dpf larvae were collected and fixed in 4% PFA/PBS overnight at 4 °C. Next, they were dehydrated with MeOH and stored at −20 °C. Larvae were rehydrated with DEPC-PBST and permeated with protease K (10 μg/ml) in DEPC-PBST. After permeation, larvae were washed with DEPC-PBST twice and then blocked in Hyb+ Buffer (50% Formamide, 5× SSCT, 0.5 mg/ml yeast RNA, 0.05 mg/ml heparin, 0.5% Tween) for 4 h at 65 °C. Next, larvae were incubated overnight at 65 °C with DIG-labeled probe in Hyb+. After probes were removed next day, samples were washed with 50% Formamide/2× SSCT for 25 min, 2× SSCT for 15 min, and 0.2× SSCT for 30 min at 65 °C. Samples were washed in MABT thrice at room temperature, blocked in blocking buffer (2% blocking reagent, 10% inactivated goat serum in MABT) for 1 h at room temperature, and then incubated with anti-DIG-AP Fab fragments in blocking buffer at 4 °C overnight. Samples were washed eight times in MABT for 30 min and three times in staining buffer (100 mM Tris-Cl pH 9.5, 100 mM NaCl, 50 mM MgCl) for 5 min. Then, larvae were stained with BM Purple AP substrate in dark. Lastly, samples were fixed with 4% PFA/PBS for 30 min and washed with PBST twice, and then were steeped in 90% glycerol.

### DNA methylation interference in zebrafish germline

#### The experiment for 5-Aza-dC treatment at 9 dpf

5-Aza-dC power (Catalog No. A3656, Sigma) was dissolved in sterile deionized water (as a stock solution of 0.25 mg/ml). Then 5-Aza-dC treatment was initiated at 9 dpf and terminated at 60 dpf in the *vasa::egfp* transgenic zebrafish. 20–25 juveniles were raised in a 1-L tank from 9 dpf to 35 dpf, then transferred to a 10-L tank till 60 dpf, and finally transferred to a zebrafish recirculation system. Fish were fed twice per day: fairy shrimp mixed with 5-Aza-dC (5 μg/g, 10 μg/g, and 15 μg/g, respectively) in the morning, and normal diet in the afternoon. Half of the water within a tank was renewed per day. The fish genders were monitored under the fluorescence microscope at 35 dpf, 60 dpf, and 90 dpf, respectively. The replicate details are shown in Table S5.

#### The experiment for aromasin + 5-Aza-dC treatment during sex transition

Aromasin tablets (Pfizer, New York, NYC) were dissolved in sterile deionized water (as a stock solution of 25 mg/ml). The *vasa::egfp* transgenic adult females (3–4 months old) with normal mating behavior and fertility were randomly selected and allocated into three groups: the control group, the aromasin-only treatment group, and the aromasin + 5-Aza-dC treatment group. 6–7 fish were put into a 1-L tank for each group. In the aromasin-only treatment group, aromasin (final concentration: 1 mg/g) was mixed with fairy shrimp for feeding. In the aromasin + 5-Aza-dC treatment group, 5-Aza-dC (final concentration: 35 μg/g) was mixed with fairy shrimp along with aromasin. Zebrafish were fed twice per day: drug food in the morning and normal diet in the afternoon. Half of the water within a tank was renewed per day.

The drug treatment was continuously performed for 3 months. Then, the fish were transferred to a zebrafish recirculation system and raised till 5 months. The ovarian fluorescence changes were monitored under the fluorescence microscope weekly till 5 months. During 4–5 months, the fish were mated with normal females to check whether they were successfully sex-reversed and fertile or not. At the 3rd month and 5th month, we also counted and calculated the sex ratios for the control group, aromasin-only treatment group, and aromasin + 5-Aza-dC treatment group, respectively. Additionally, for each stage during sex transition, some of the gonads were collected for photographing and histological staining and the remaining gonads were subjected to DNA methylation and RNA library construction. The replicate details for the treatment experiment are shown in Table S5.

### Isolation and collection of zebrafish samples during female-to-male sex transition

The gonad was isolated from each individual fish during aromasin treatment and then separated from surrounding tissue and blood in D-hanks solution. The gonad was divided into two parts: one was immediately frozen at −80 °C in RNAlater (Catalog No. R0901, Sigma) to be used for RNA-seq, and the other was immediately frozen at −80 °C in PBS for DNA extraction.

Wild-type and sex-reversed sperm were released from testes by gently pipetting in Hank’s balanced salt solution (HBSS) for 15 min at 28 °C. Then, the supernatant with swimming sperm was transferred to a new tube. The incubation-transfer was repeated 3 times and finally pelleted by centrifugation for 5 min at 10,000 *g*. 100 μl Buffer X2 cell lysis solution (20 mM Tris-HCl pH 8.0, 20 mM EDTA, 200 mM NaCl, 80 mM DTT, 4% SDS, 250 μg/ml Proteinase K) was added to the samples, and incubated at 55 °C until the samples were dissolved (at least 1 h) on a rocking platform.

### Bisulfate-seq library preparation for zebrafish sex transition samples

Samples (including wild-type/sex-reversed sperm and gonads) were subjected to genomic DNA extraction (>100 ng) using QIAamp DNA Mini Kit (Catalog No. 51304, Qiagen, Hilden, Germany) following the manufacturer’s protocol. Purified DNA spiked in with 0.5% un-methylated lambda DNA was sonicated into 300 bp fragments with Covaris S220. Sheared DNA was transferred to a fresh PCR tube. NEBNext Ultra II DNA Library Prep Kit for Illumina was used for library construction according to manufacturer’s instruction and as described above. Then bisulfite conversion was performed using the EZ DNA Methylation-Gold Kit according to the instruction manual. Bisulfite-treated DNA was amplified using KAPA HiFi HotStar Uracil + ReadyMix with 9–10 cycles. 300–500 bp DNA fragments were selected using Ampure XP beads with 0.5× volume plus 0.65× volume, then eluted in 15 μl ddH_2_O. DNA methylation libraries were sequenced on Hiseq2000 or Hiseq Xten platform (Illumina)

### RNA-seq for zebrafish gonads and gametes

Zymo Quick-RNA MicroPrep Kit (Catalog No. R1050, Zymo Research) was used to extract RNA according to manufacturer’s protocol. RNA quality was assessed on agarose gel. The NEBNext Poly (A) mRNA Magnetic Isolation Module (Catalog No. E7490, NEB) was used to isolate intact poly (A)^+^ RNA from the 200–800 ng total RNA followed by reverse transcription and amplification. Further, library was prepared using NEBNext Ultra II directional RNA Library Prep Kit (Catalog No. E7760, NEB) according to the manufacturer’s instructions. Libraries were pooled and sequenced on a Hiseq Xten platform with PE150 mode (Illumina).

### DNA methylation data processing

Raw reads were trimmed to remove the adapter-containing and low-quality reads by Trimmomatic software with default parameters [55]. Clean reads were aligned by using Bismark (version 12.5) [56] against zebrafish genome assembly (Zv9) in paired-end mode with parameters: -N 1 –X 600. The lambda genome was also included in the reference sequence as an extra chromosome for bisulfite conversion rate calculation. After alignment, overlapping part of paired reads was clipped using ‘clipOverlap’ function of BamUtil (https://genome.sph.umich.edu/wiki/BamUtil:_clipOverlap). PCR duplications were removed with Picard (http://broadinstitute.github.io/picard). CpG methylation calls were extracted with ‘Bismark methylation extractor’ function. Strands were merged to calculate the CpG methylation level per site. aML at each stage was the mean of methylation level at each CpG site. The CpG density was defined as the average number of CpG sites per 100 bp.

Genomic elements were downloaded from UCSC table browser. The aML of genomic elements was measured as the sum of the methylation levels of CpGs divided by the total number of CpGs that reside in those genomic elements.

The R package ‘ape’ was used to perform hierarchical clustering analysis with ‘euclidean’ distance by the ‘complete’ clustering method (500 bp bin for each unit). The R package methylKit [57] was used to cluster sex transition samples hierarchically with ‘euclidean’ distance by the ‘ward’ clustering method (500 bp bin for each unit).

The DNA methylation profiles of zebrafish gametes and embryos were downloaded from the Gene Expression Omnibus database (GEO: GSE44075).

### Identification of DMCs and DMRs

To detect DMCs (differentially methylated cytosines), ‘mcomp’ module from R package MOABS [58] was employed. CpGs with *P* value < 0.01 and methylation level difference between two stages > 0.2 were considered as DMCs. DMRs were identified by a smoothing local likelihood method in R package bsseq [59]. Regions which contain at least five DMCs and have methylation level difference between two stages > 0.2 were defined as DMRs.

### Identification of DMPs

The methylation level of each promoter was determined as the ratio of the number of alignments with C (methylated) over the sum of alignments with C and T for all CpGs in the promoter. DMPs were identified with two-tailed Fisher’s exact test, and *P* values were adjusted by Benjamini–Hochberg method. Promoters with adjusted *P* value < 0.01 and methylation level difference > 0.2 were considered as DMPs. DMP clustering for zebrafish PGCs was performed using the ‘pam’ function of the ‘fpc’ package in R.

### RNA-seq data processing

Reads with low-quality and adapters were trimmed by Trimmomatic software and then aligned by using STAR (version 2.5.2b) with default parameters [60]. Then filtering was performed to remove alignments with MAPQ < 20. The unique reads were used to calculate the fragments per kilobase of transcript per million fragments mapped (FPKM) with Cufflinks (version 2.2.1) (http://cufflinks.cbcb.umd.edu). We filtered out genes with FPKM < 1 in all cell types. RNA-seq tracks for visualization were generated by ‘bamCoverage’ tool in Deeptools2 with parameter: --normalizeUsingRPKM.

### Time-course gene expression analysis

To analysis transient gene expression changes and to study the dynamics of their transcriptional activity from ovary to testis, we performed time-course sequencing data analysis by using R package TC-seq (version 1.9) with default parameters. We selected genes with top 50% expression variance and membership value > 0.4 for further analysis.

### GO analysis

GO analysis for genes with DMPs was performed using DAVID [61]. GO analysis for genes in different time-course clusters was performed by Metascape (http://metascape.org) [62]. GO terms with *P* < 0.05 (Fisher’s exact test) were considered statistically significant.

### Statistical analysis

R and Prism were used for statistical analyses. Pearson’s correlation coefficient was calculated using the ‘cor.test’ function with default parameters. One-way ANOVA was used for statistical analysis in the DNA methylation inhibitor (5-Aza-dC) treatment experiment. Unpaired two-sided *t*-test was used for statistical analysis in the drug treatment experiment (aromasin-only and aromasin + 5-Aza-dC). For significance marks, *, *P* < 0.05; **, *P* < 0.01; ***, *P* < 0.001.

## Data availability

The sequence data generated in this study have been deposited in the Genome Sequence Archive [63] at the National Genomics Data Center, Beijing Institute of Genomics, Chinese Academy of Sciences / China National Center for Bioinformation (GSA: CRA002472), and are publicly accessible at https://bigd.big.ac.cn/gsa/.

## CRediT author statement

**Xinxin Wang:** Conceptualization, Software, Validation, Investigation, Writing - original draft. **Xin Ma:** Validation, Investigation. **Gaobo Wei:** Validation, Resources. **Weirui Ma:** Investigation, Resources. **Zhen Zhang:** Resources. **Xuepeng Chen:** Software, Formal analysis, Investigation. **Lei Gao:** Validation. **Zhenbo Liu:** Investigation. **Yue Yuan:** Investigation. **Lizhi Yi:** Investigation. **Jun Wang:** Resources. **Toshinobu Tokumoto:** Resources. **Junjiu Huang:** Resources, Funding acquisition. **Dahua Chen:** Resources, Supervision. **Jian Zhang:** Resources, Writing - review & editing, Supervision. **Jiang Liu:** Conceptualization, Writing - review & editing, Project administration, Funding acquisition, All authors read and approved the final manuscript.

## Competing interests

The authors have declared no competing interests.

## Supplementary material

Supplementary Figure S1 Isolation of PGCs and germ cells in zebrafish A. The left panel shows the fluorescence images of immature ovary and immature testis at 35 dpf; the right panel shows the H&E staining of immature ovary and immature testis at 35 dpf. Females and males with a body length of 1.7 ± 0.1 cm were selected. The scale bar of left panel is 1 mm. The scale bar of right panel is 50 µm. **B.** Sorting germ cells by FACS. Left panel shows GFP-negative cells for control fish. Right panel shows GFP-positive cells for two transgenic zebrafish strains (*kop-gfp-nos1-3′UTR* and *vasa::egfp*).

Supplementary Figure S2 The DNA methylation dynamics for different genomic elements in zebrafish germ cells A. Boxplots show the correlation between CpG density and methylation level in TSS ± 2 kb regions. Boxes and whiskers represent the 25th/50th/75th percentiles and 1.5 x the interquartile range, respectively. **B.** Proportion of CpGs with high methylation level (ML ≥ 0.75), intermediate methylation level (0.25 < ML < 0.75), and low methylation level (ML ≤ 0.25) for indicated genomic features: 5′UTRs (untranslated regions), exons, introns, 3′UTRs, gene body, intergenic regions, and CGIs (CpG islands). **C.** Proportion of CpGs with high methylation level, intermediate methylation level, and low methylation level for indicated genomic features: repeat elements and noncoding RNAs.

Supplementary Figure S3 Transcriptional characteristics of zebrafish PGCs A. Heatmap for stage-specific gene expression from 4 hpf PGCs to 35 dpf germ cells. 6 clusters were identified by k-means method. The right panels show the corresponding cluster-enriched GO biological process terms by using DAVID. *P* values are also shown. **B.** Bar plots for the gene expression levels of germ cell-related genes at different stages. Gene expression levels are averaged from two biological replicates.

Supplementary Figure S4 **DNA methylation dynamics at promoter regions across zebrafish germ****cell****development A.** Snapshot for the DNA methylation of 9 dpf-specific hypomethylated promoter gene *myoc*. Dynamic regions around promoters are highlighted in gray. DNA methylation level ranges from 0 to 1. Each vertical line represents one CpG site. **B.** Snapshot for the DNA methylation of 9 dpf-specific hypomethylated promoter gene *tdrd1*. **C.** Snapshot for the DNA methylation of 9 dpf-specific hypomethylated promoter gene *ta*.

Supplementary Figure S5 **The tet3 morpholino knock down can influence the zebrafish PGC development A.** Boxplots of CpG methylation level in DMRs (differentially methylated regions) identified by pairwise comparisons between adjacent stages. The hypermethylated stage is denoted as red box, the hypomethylated stage is denoted as blue box, and the rest of stages is denoted as gray box. Boxes and whiskers represent the 25th/50th/75th percentiles and 1.5 x the interquartile range, respectively. N represents the number of DMRs. **B.** The Western blot for *tet3* in WT larvae, control MO larvae, and *tet3* MO larvae at 3 dpf. Tubulin is the loading control. **C.** The images for WT larvae, *tet1* MO larvae, tet3 MO larvae, and *tet1/3* MO larvae at 3 dpf under microscopy. WT, wild type. **D.** The fluorescence images for WT and *tet3* MO germ cells at 5 dpf and 9 dpf (red box). **E.** Dot plot showing changes in the number of PGCs at 5 dpf (WT, *n* = 22; *tet3* MO, *n* = 27) and 9 dpf (WT, *n* = 22; *tet3* MO, *n* = 23), which was counted by the squash method. The data represents mean ± SD. The P value was calculated by *t*-test. *, *P* < 0.05. **, *P* < 0.01, ***, *P* < 0.001.

Supplementary Figure S6 **F**-**t**-**male sex transition involves functional changes and extensive DNA reprogramming A.** Embryos generated from SR fish mated with WT female. **B.** Hierarchical clustering analysis according to the DNA methylation pattern for the biological replicates of ovary, gonads from zebrafish treated with aromasin for 1 month, gonads from zebrafish treated with aromasin for 2 months, gonads from zebrafish treated with aromasin for 3 months, SR testis, and WT testis. The R package methylKit was used to cluster sex transition samples hierarchically with ‘euclidean’ distance by the ‘ward’ clustering method (500 bp bin for each unit). **C.** Bimodal distribution of CpG methylation level across the genome for zebrafish at the indicated stages: ovary, 1 month, 2 months and 3 months, SR testis, and WT testis. **D.** Snapshot of DNA methylation tracks for *dnmt6* in oocyte, WT sperm, and SR sperm. Dynamic regions around the promoter are highlighted in gray. DNA methylation level ranges from 0 to 1. Each vertical line represents one CpG site. **E.** Snapshot of DNA methylation tracks for *sycp3l* in oocyte, WT sperm, and SR sperm. SR, sex-reversed. WT, wild-type.

Supplementary Figure S7 **DNA methylation is related to the expression of sex determination genes in zebrafish A.** DMPs between testis and ovary. GO terms for the testis-specific hypomethylated and ovary-specific hypomethylated DMPs are shown in the right panel. Promoters are defined as regions 1000 bp upstream and downstream of TSSs (transcriptional start sites) for each gene. **B.** Heatmap shows the methylation level of DMPs between ovary and testis for key sex determination genes. **C.** Snapshot of DNA methylation and gene expression tracks for the sex determination gene *cyp19a1a* in ovary, 1 month, 2 months, 3 months, SR testis, and WT testis. **D.** The comparison of DNA methylation reprogramming during germ cell development process (left panel) and sex transition process (right panel) in zebrafish. **E.** The heatmap shows the dynamic changes of DMPs during germ cell development process and sex transition process (DMPs, *n* = 574).

Supplementary Table S1 **DNA methylation data summary for zebrafish germ****cell****and sex****transition****samples**

Supplementary Table S2 **Transcriptome data summary for zebrafish germ****cell and sex****transition****samples**

Supplementary Table S3 **GO terms for stage-specific genes in zebrafish germ cells**

Supplementary Table S4 **GO terms for differentially methylated promoters (DMPs) in zebrafish germ****cells**

Supplementary Table S5 **Sex ratio summary for drug treatment fish**

Supplementary Table S6 **GO terms for time-course clustering genes during zebrafish sex transition**

Supplementary Table S7 **GO terms for DMPs between testis and ovary**
