# Supplementary figures and images for: The Role of DNA Methylation Reprogramming During Sex Determination and Transition in Zebrafish
Source: Genomics Proteomics Bioinformatics. 2021 Feb 19;19(1):48–63. doi: 10.1016/j.gpb.2020.10.004 (PMC8640932; doi:10.1016/j.gpb.2020.10.004)

**A**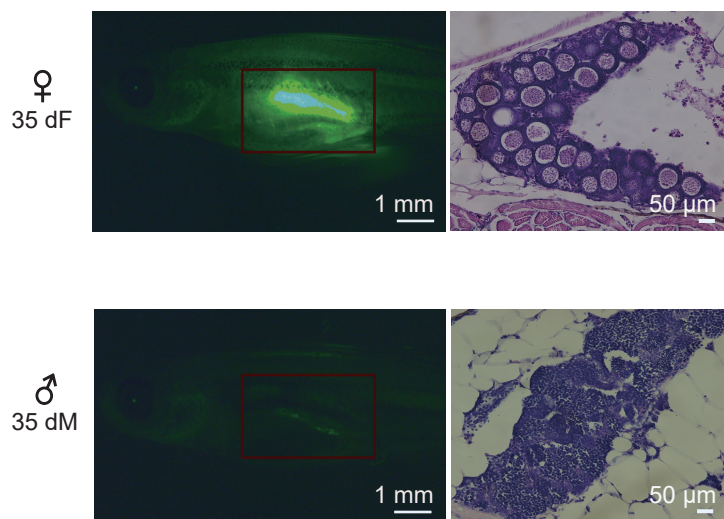**B**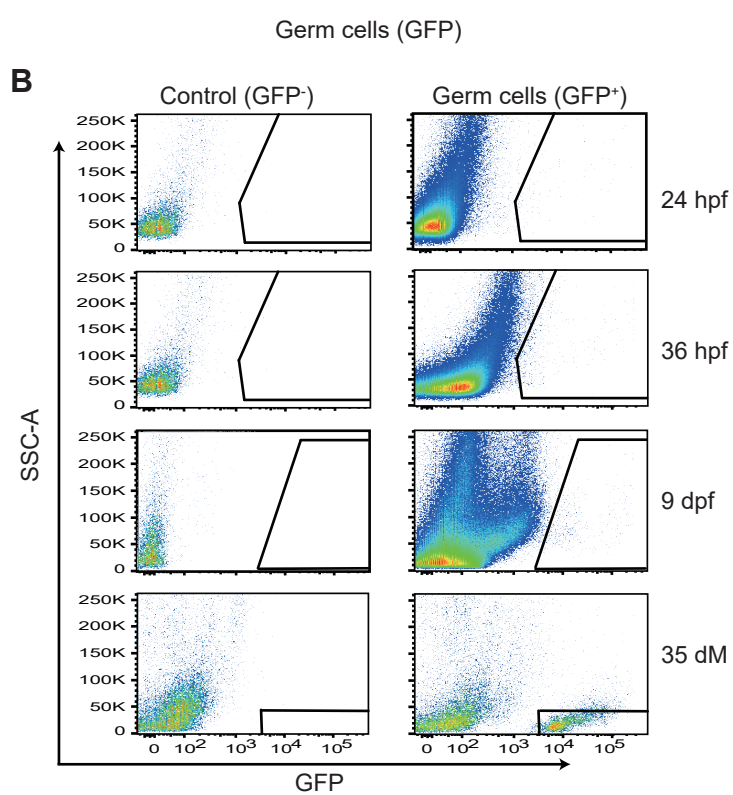

Supplement: Supplementary Figure S1 — Isolation of PGCs and germ cells in zebrafish A. The left panel shows the fluorescence images of immature ovary and immature testis at 35 dpf; the right panel shows the H&E staining of immature ovary and immature testis at 35 dpf. Females and males with a body length of 1.7 ± 0.1 cm were selected. The scale bar of left panel is 1 mm. The scale bar of right panel is 50 µm. B. Sorting germ cells by FACS. Left panel shows GFP-negative cells for control fish. Right panel shows GFP-positive cells for two transgenic zebrafish strains (kop-gfp-nos1-3′UTR and vasa::egfp). [file mmc1.pdf]

**A**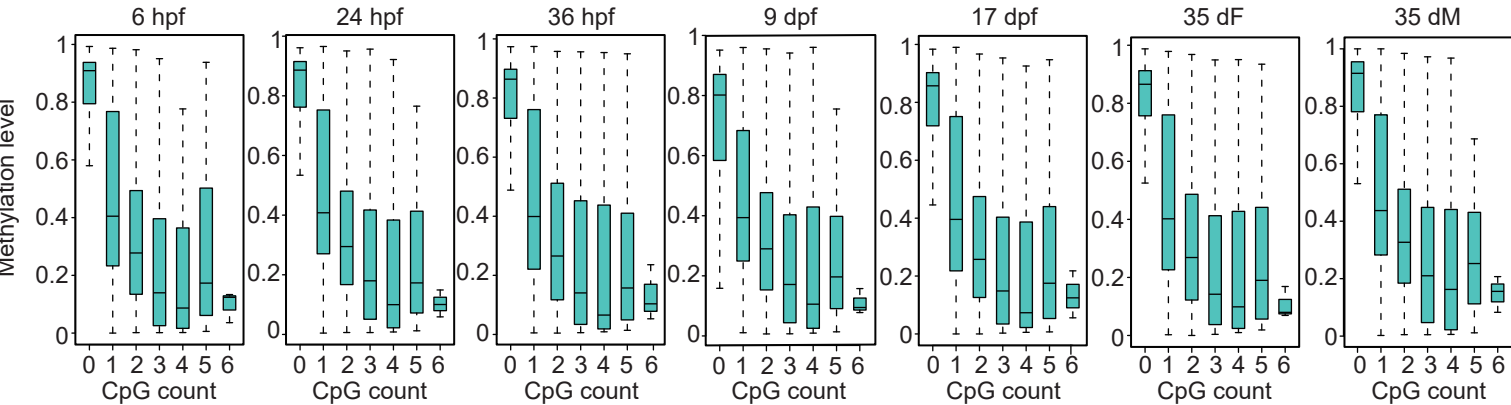**B**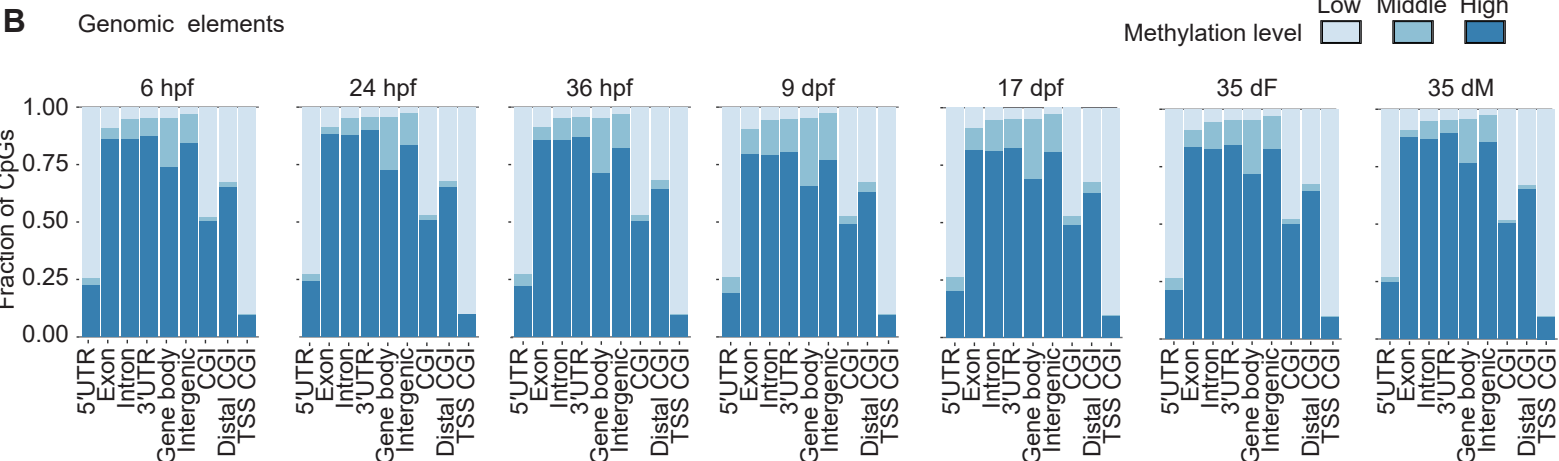**C**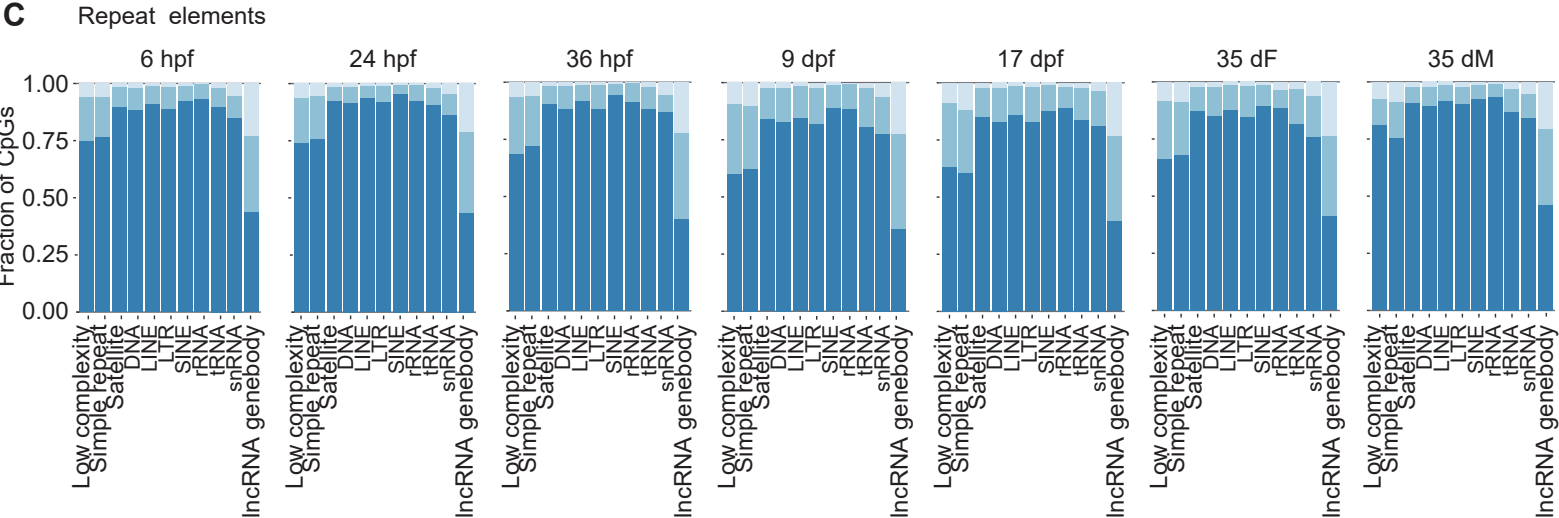

Supplement: Supplementary Figure S2 — The DNA methylation dynamics for different genomic elements in zebrafish germ cells A. Boxplots show the correlation between CpG density and methylation level in TSS ± 2 kb regions. Boxes and whiskers represent the 25th/50th/75th percentiles and 1.5 x the interquartile range, respectively. B. Proportion of CpGs with high methylation level (ML ≥ 0.75), intermediate methylation level (0.25 < ML < 0.75), and low methylation level (ML ≤ 0.25) for indicated genomic features: 5′UTRs (untranslated regions), exons, introns, 3′UTRs, gene body, intergenic regions, and CGIs (CpG islands). C. Proportion of CpGs with high methylation level, intermediate methylation level, and low methylation level for indicated genomic features: repeat elements and noncoding RNAs. [file mmc2.pdf]

**A**

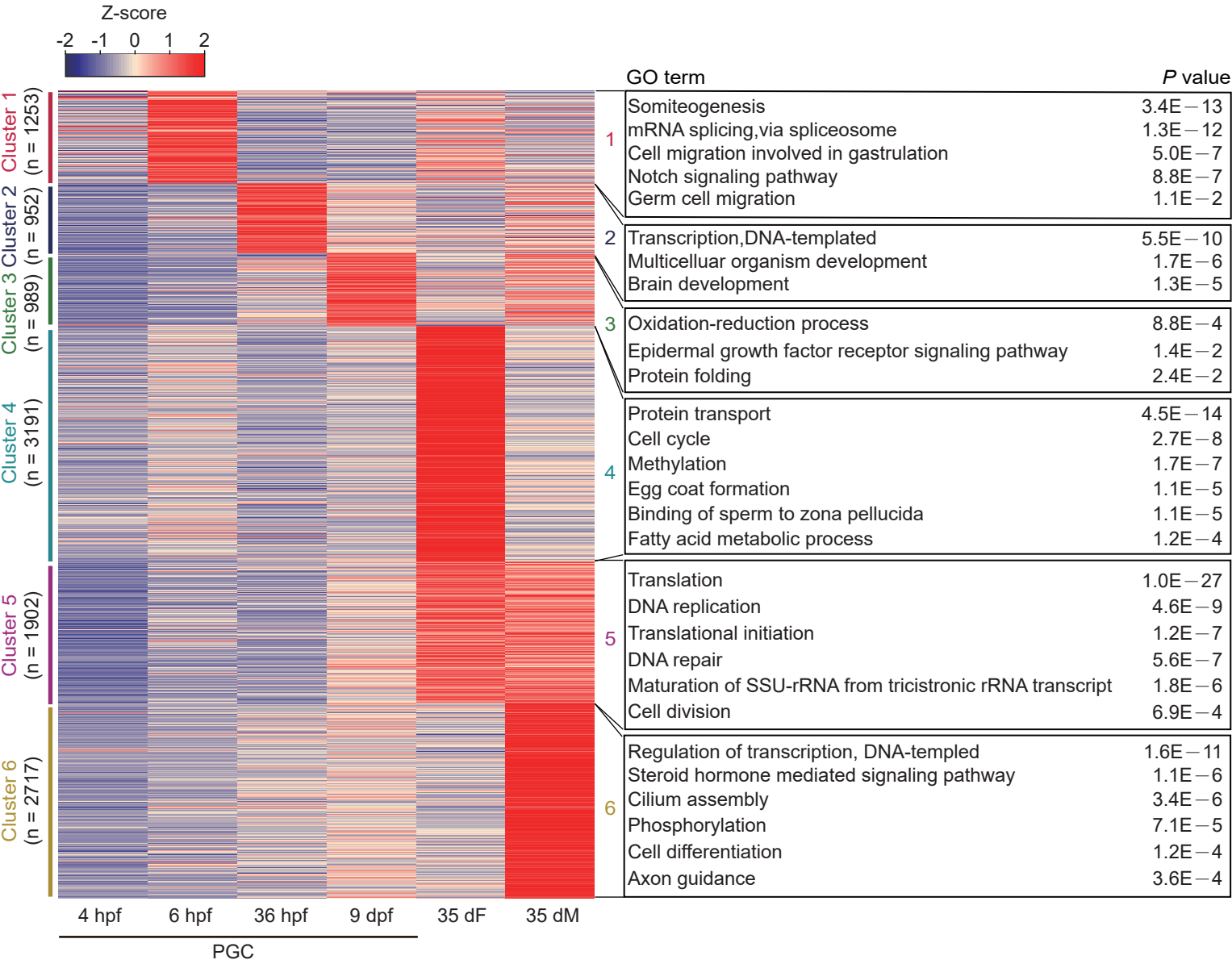

**B**

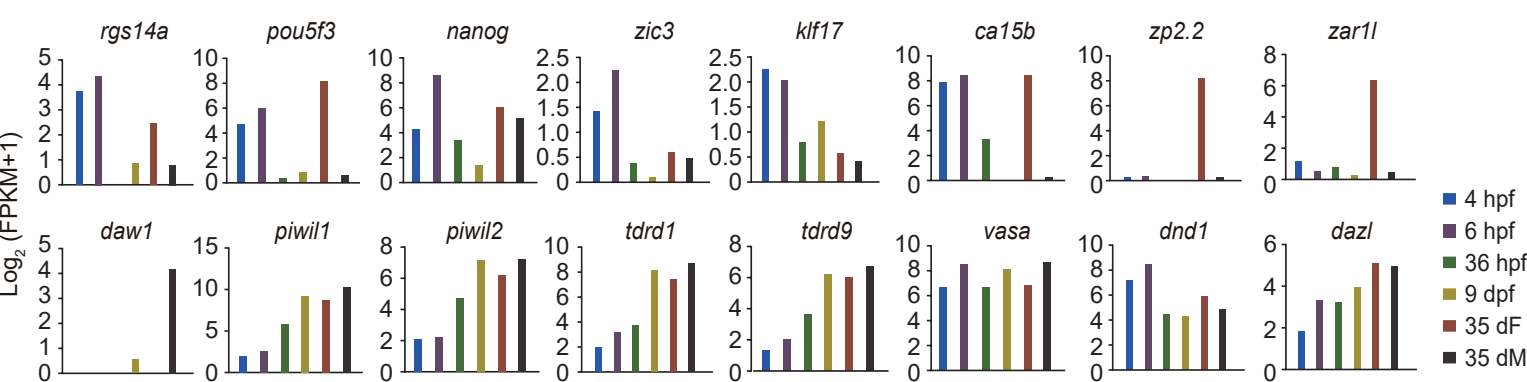

Supplement: Supplementary Figure S3 — Transcriptional characteristics of zebrafish PGCs A. Heatmap for stage-specific gene expression from 4 hpf PGCs to 35 dpf germ cells. 6 clusters were identified by k-means method. The right panels show the corresponding cluster-enriched GO biological process terms by using DAVID. P values are also shown. B. Bar plots for the gene expression levels of germ cell-related genes at different stages. Gene expression levels are averaged from two biological replicates. [file mmc3.pdf]

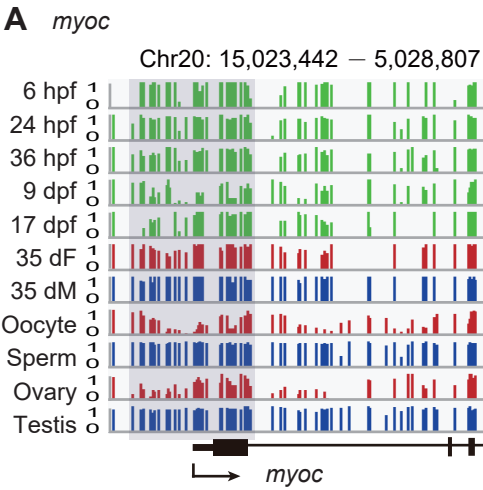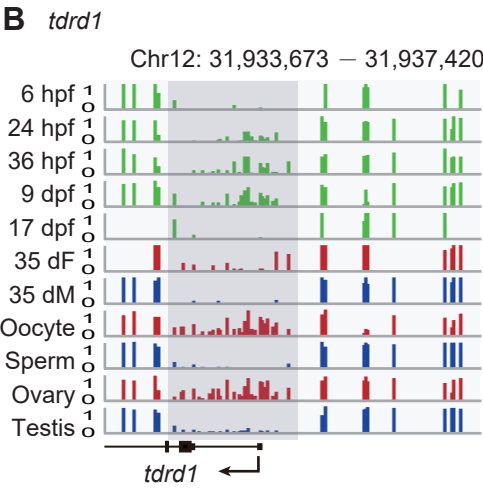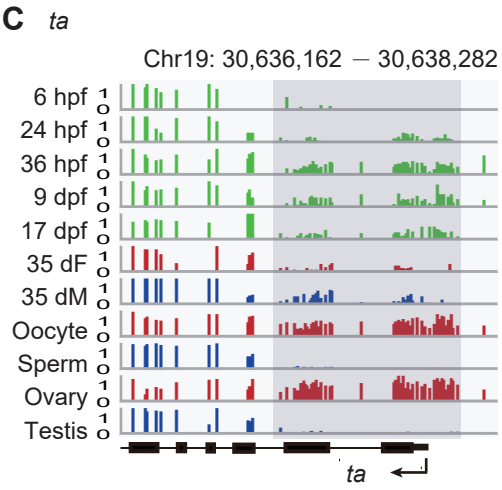

Supplement: Supplementary Figure S4 — DNA methylation dynamics at promoter regions across zebrafish germcelldevelopment A. Snapshot for the DNA methylation of 9 dpf-specific hypomethylated promoter gene myoc. Dynamic regions around promoters are highlighted in gray. DNA methylation level ranges from 0 to 1. Each vertical line represents one CpG site. B. Snapshot for the DNA methylation of 9 dpf-specific hypomethylated promoter gene tdrd1. C. Snapshot for the DNA methylation of 9 dpf-specific hypomethylated promoter gene ta. [file mmc4.pdf]

**A**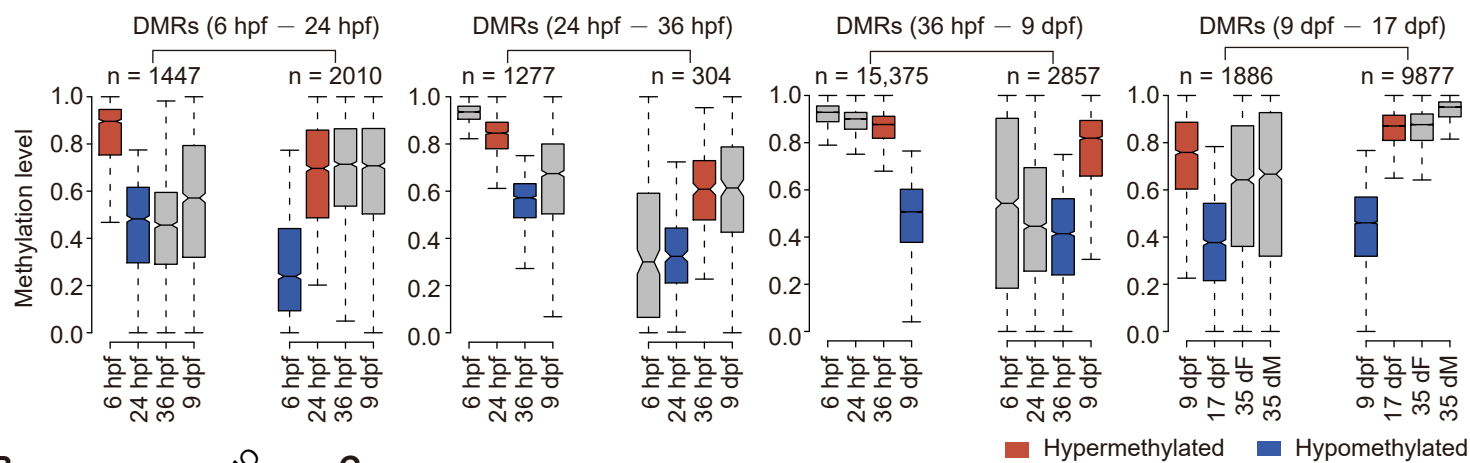**B**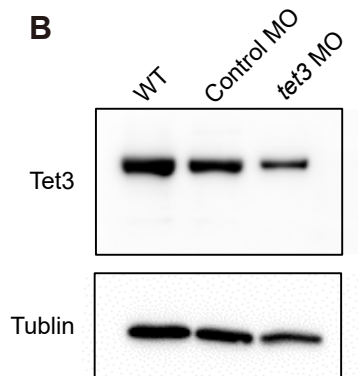**C**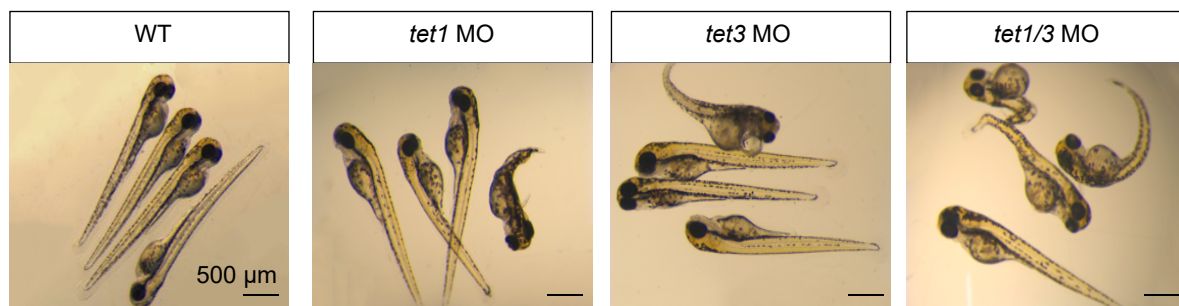**D**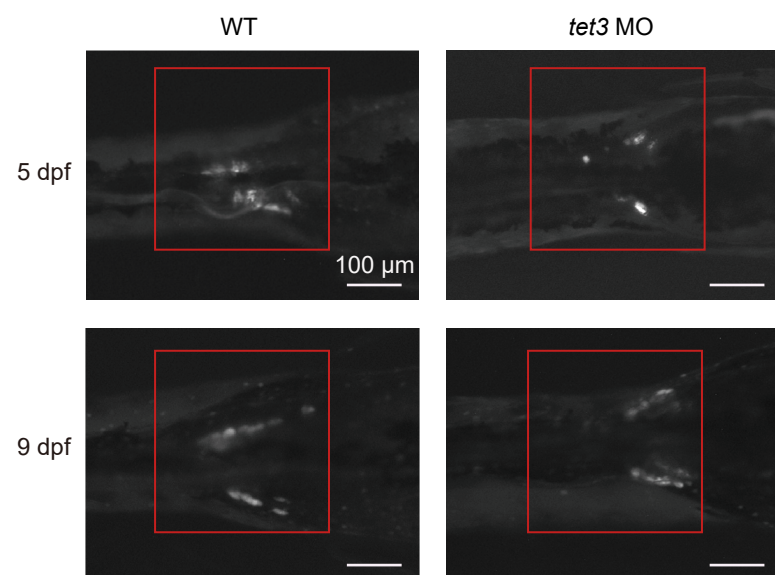**E**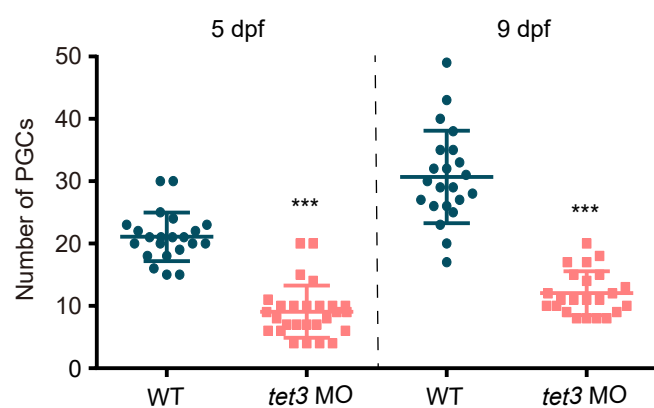

Supplement: Supplementary Figure S5 — The tet3 morpholino knock down can influence the zebrafish PGC development A. Boxplots of CpG methylation level in DMRs (differentially methylated regions) identified by pairwise comparisons between adjacent stages. The hypermethylated stage is denoted as red box, the hypomethylated stage is denoted as blue box, and the rest of stages is denoted as gray box. Boxes and whiskers represent the 25th/50th/75th percentiles and 1.5 x the interquartile range, respectively. N represents the number of DMRs. B. The Western blot for tet3 in WT larvae, control MO larvae, and tet3 MO larvae at 3 dpf. Tubulin is the loading control. C. The images for WT larvae, tet1 MO larvae, tet3 MO larvae, and tet1/3 MO larvae at 3 dpf under microscopy. WT, wild type. D. The fluorescence images for WT and tet3 MO germ cells at 5 dpf and 9 dpf (red box). E. Dot plot showing changes in the number of PGCs at 5 dpf (WT, n = 22; tet3 MO, n = 27) and 9 dpf (WT, n = 22; tet3 MO, n = 23), which was counted by the squash method. The data represents mean ± SD. The P value was calculated by t-test. *, P < 0.05. **, P < 0.01, ***, P < 0.001. [file mmc5.pdf]

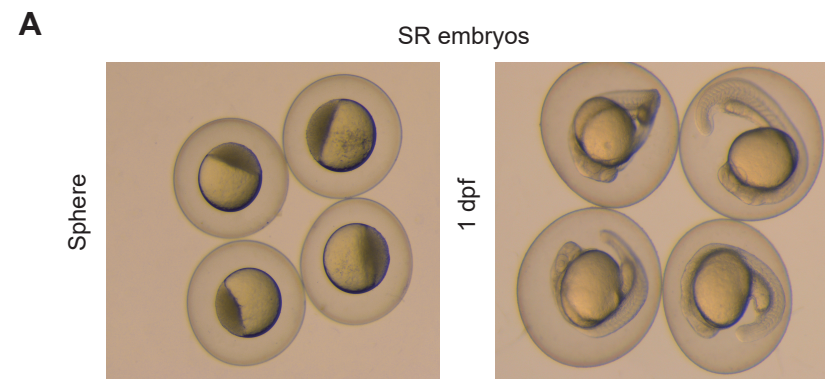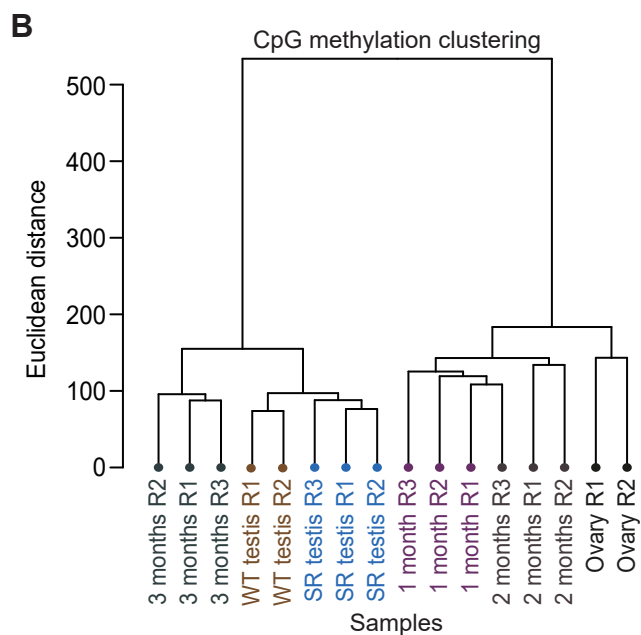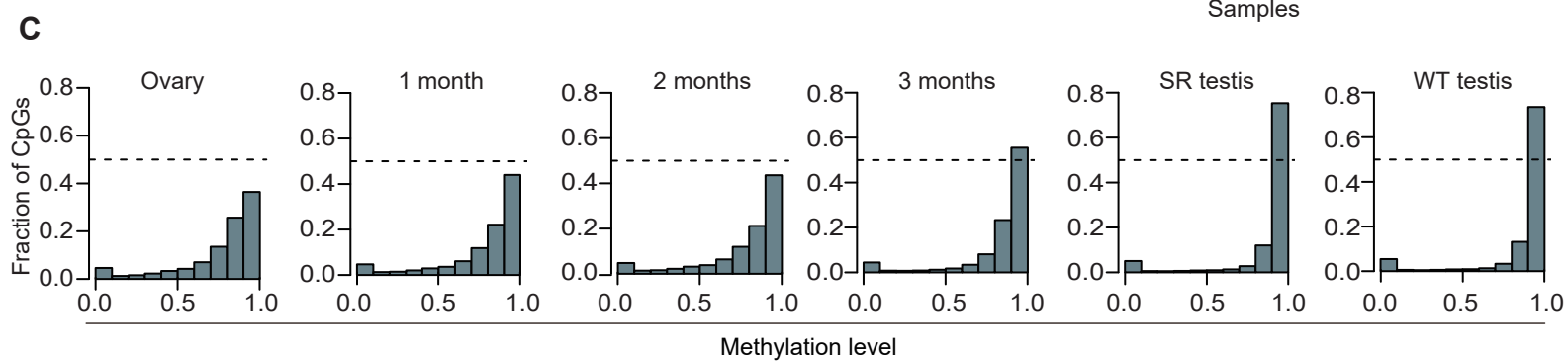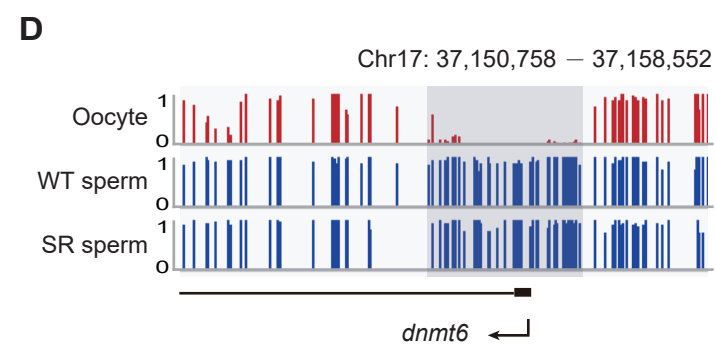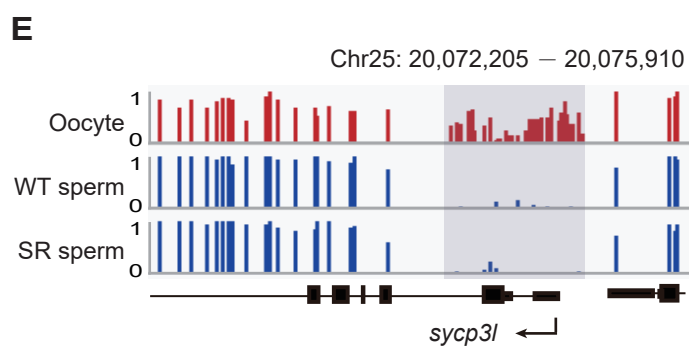

Supplement: Supplementary Figure S6 — F-t-male sex transition involves functional changes and extensive DNA reprogramming A. Embryos generated from SR fish mated with WT female. B. Hierarchical clustering analysis according to the DNA methylation pattern for the biological replicates of ovary, gonads from zebrafish treated with aromasin for 1 month, gonads from zebrafish treated with aromasin for 2 months, gonads from zebrafish treated with aromasin for 3 months, SR testis, and WT testis. The R package methylKit was used to cluster sex transition samples hierarchically with ‘euclidean’ distance by the ‘ward’ clustering method (500 bp bin for each unit). C. Bimodal distribution of CpG methylation level across the genome for zebrafish at the indicated stages: ovary, 1 month, 2 months and 3 months, SR testis, and WT testis. D. Snapshot of DNA methylation tracks for dnmt6 in oocyte, WT sperm, and SR sperm. Dynamic regions around the promoter are highlighted in gray. DNA methylation level ranges from 0 to 1. Each vertical line represents one CpG site. E. Snapshot of DNA methylation tracks for sycp3l in oocyte, WT sperm, and SR sperm. SR, sex-reversed. WT, wild-type. [file mmc6.pdf]

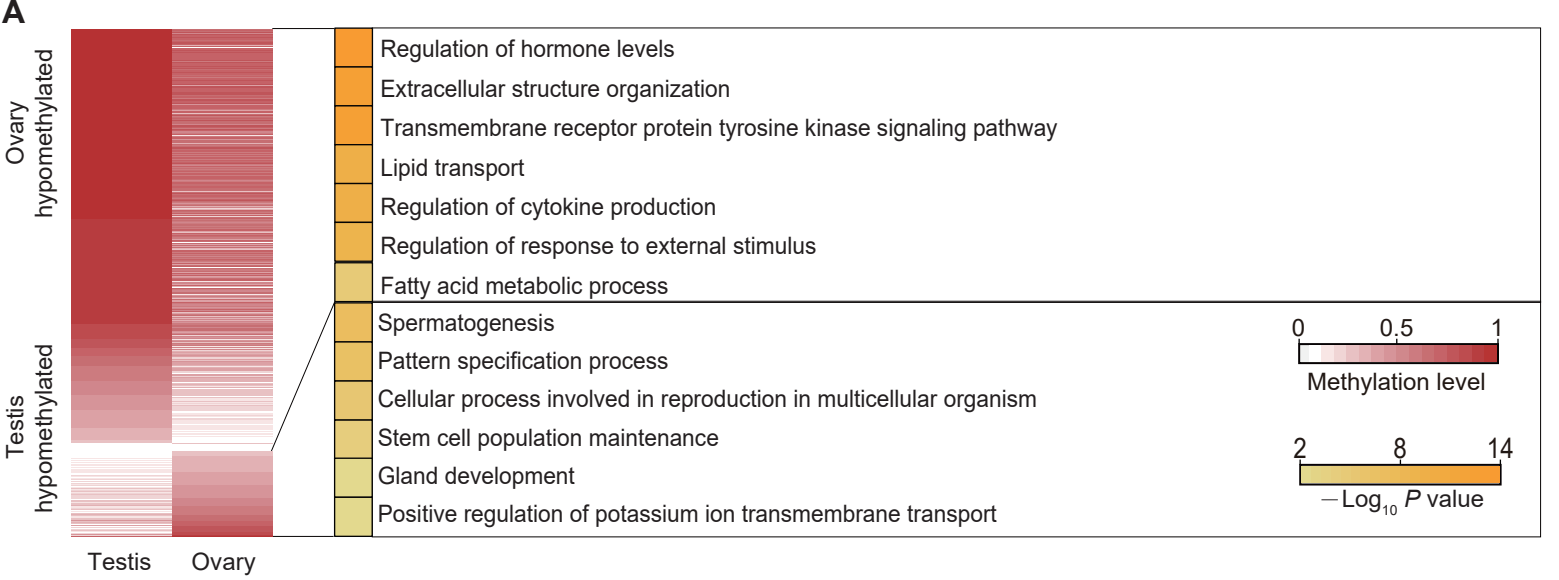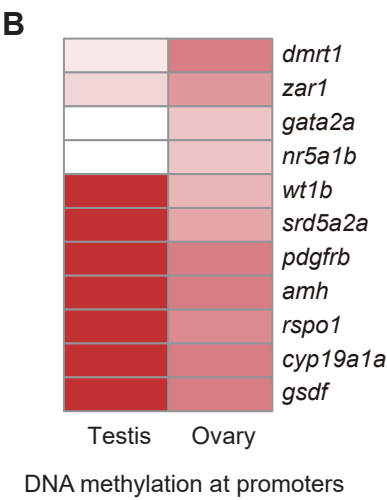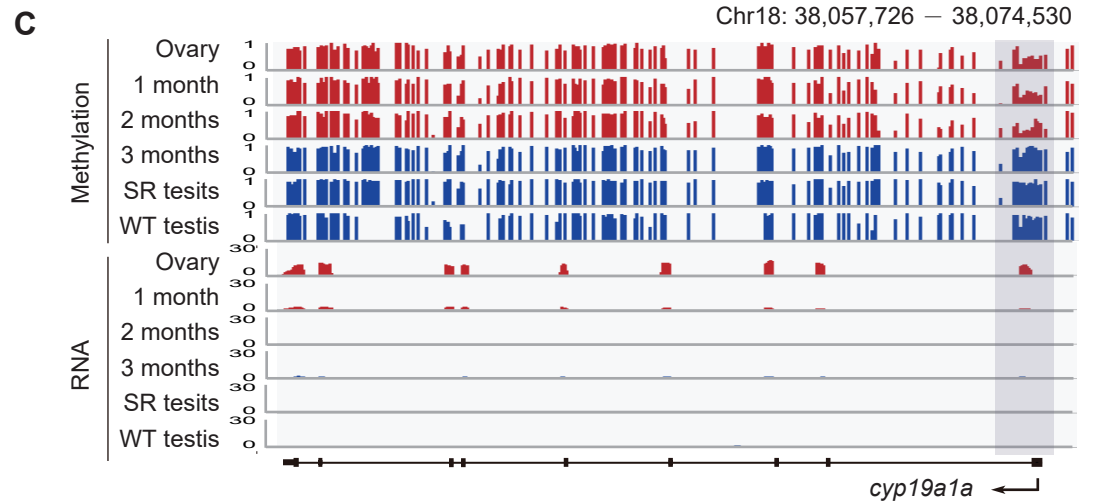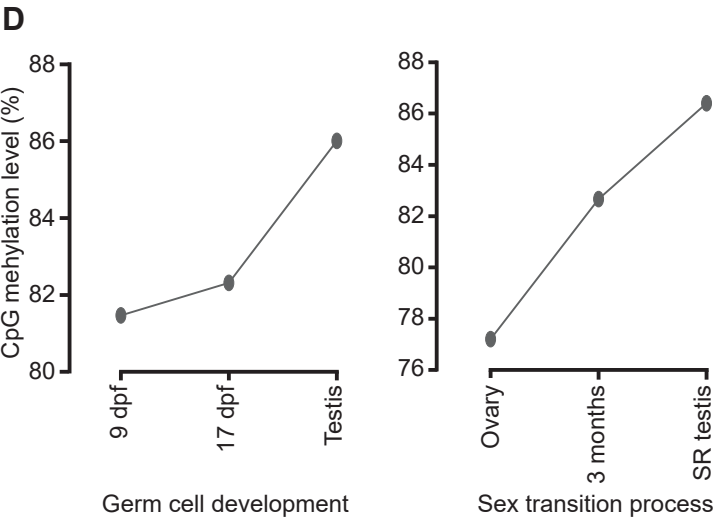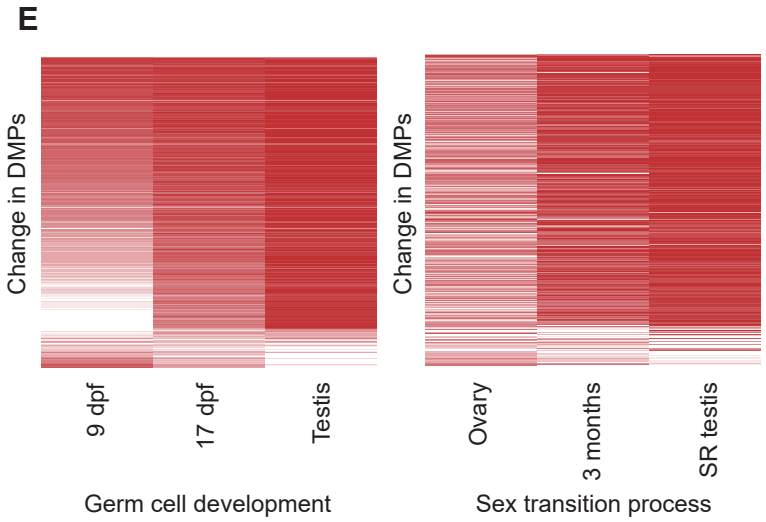

Supplement: Supplementary Figure S7 — DNA methylation is related to the expression of sex determination genes in zebrafish A. DMPs between testis and ovary. GO terms for the testis-specific hypomethylated and ovary-specific hypomethylated DMPs are shown in the right panel. Promoters are defined as regions 1000 bp upstream and downstream of TSSs (transcriptional start sites) for each gene. B. Heatmap shows the methylation level of DMPs between ovary and testis for key sex determination genes. C. Snapshot of DNA methylation and gene expression tracks for the sex determination gene cyp19a1a in ovary, 1 month, 2 months, 3 months, SR testis, and WT testis. D. The comparison of DNA methylation reprogramming during germ cell development process (left panel) and sex transition process (right panel) in zebrafish. E. The heatmap shows the dynamic changes of DMPs during germ cell development process and sex transition process (DMPs, n = 574). [file mmc7.pdf]
